# A Peptide Link Between Human Cytomegalovirus Infection, Neuronal Migration, and Psychosis

**DOI:** 10.3389/fpsyt.2020.00349

**Published:** 2020-05-08

**Authors:** Guglielmo Lucchese, Agnes Flöel, Benjamin Stahl

**Affiliations:** ^1^ Department of Neurology, University of Greifswald, Greifswald, Germany; ^2^ Department of Computing, Goldsmiths, University of London, London, United Kingdom; ^3^ Partner Site Rostock/Greifswald, German Center for Neurodegenerative Diseases, Greifswald, Germany; ^4^ Department of Neurology, Charité Universitätsmedizin Berlin, Berlin, Germany; ^5^ Department of Neurophysics, Max Planck Institute for Human Cognitive and Brain Sciences, Leipzig, Germany; ^6^ Psychologische Hochschule Berlin, Berlin, Germany

**Keywords:** peptide sharing, HCMV, immune response, schizophrenia, cross-reactivity

## Abstract

Alongside biological, psychological, and social risk factors, psychotic syndromes may be related to disturbances of neuronal migration. This highly complex process characterizes the developing brain of the fetus, the early postnatal brain, and the adult brain, as reflected by changes within the subventricular zone and the dentate gyrus of the hippocampus, where neurogenesis persists throughout life. Psychosis also appears to be linked to human cytomegalovirus (HCMV) infection. However, little is known about the connection between psychosis, HCMV infection, and disruption of neuronal migration. The present study addresses the hypothesis that HCMV infection may lead to mental disorders through mechanisms of autoimmune cross-reactivity. Searching for common peptides that underlie immune cross-reactions, the analyses focus on HCMV and human proteins involved in neuronal migration. Results demonstrate a large overlap of viral peptides with human proteins associated with neuronal migration, such as ventral anterior homeobox 1 and cell adhesion molecule 1 implicated in GABAergic and glutamatergic neurotransmission. The present findings support the possibility of immune cross-reactivity between HCMV and human proteins that—when altered, mutated, or improperly functioning—may disrupt normal neuronal migration. In addition, these findings are consistent with a molecular and mechanistic framework for pathological sequences of events, beginning with HCMV infection, followed by immune activation, cross-reactivity, and neuronal protein variations that may ultimately contribute to the emergence of mental disorders, including psychosis.

## Introduction

Newly generated neurons migrate from their site of origin to specific brain areas and subregions, a process that involves adaptation with different degrees of complexity (1, 2). The cytoskeleton is regulated at the molecular and cellular level to execute neuronal migration (3); polarity in migrating neurons is reached by re-purposing of cytokinetic processes (4, 5); and blood vessels are used as a physical substrate (6). Cell adhesion, cell cycle, and angiogenesis are implicated in neuronal migration.

Clinically, disruption of this process has been related not only to severe malformations of cortical development (lissencephaly, schizencephaly, neuronal eterotopia, polymicrogyria) (7) but also to psychosis (8–10). However, the relationship between macro- and microscopic structural brain anomalies and psychosis appears to be unclear, and disruption of cellular function has been hypothesized (11, 12). According to current opinion, more subtle alterations starting early during neurodevelopment can alter neural circuits and induce psychotic syndromes during adolescence or young adulthood (13). Indeed, altered migration and development of GABAergic cortical interneurons have been linked not only to schizophrenia but also to depression and anxiety disorders and seem to be strongly dependent on other neurotransmitter networks, such as dopaminergic and glutamatergic systems (14–16).

The present study focuses on HCMV infection as a potential link between neuronal migration and psychosis. On the one hand, it has been shown that herpesvirus infection of the developing brain can disturb migration of neuronal cells in animal models (17–19). On the other hand, HCMV has been discussed in the context of psychosis. Indeed, previous research has demonstrated that maternal HCMV infection and antibodies are associated with psychosis in the offspring (20), that infection during childhood is a risk factor for later psychosis (21), and that concurrent antibody titers are associated with psychosis-related symptoms (22–25). Epidemiological evidence is then not only suggestive of an association between HCMV and psychosis but also points to an influence of the infection on the early development of the central nervous system, possibly on neuronal migration.

Therefore, we here tried to elucidate the associations between HCMV infection, aberrant neuronal migration, and psychosis, building on previous research that had assessed peptide commonality and potential immune cross-reactivity between microbial and human proteins (26–31). More specifically, we investigated the peptide platform shared by HCMV and human proteins involved in neuronal migration.

## Methods

A set formed by primary amino acid (aa) sequences of human proteins involved in neuronal migration was retrieved from the UniProtKB Database (www.uniprot.org) (32). The protein library was obtained by separately searching for “neuron” AND “migration” as well as “neuronal” AND “migration” within the *Homo sapiens* proteins in the reviewed and annotated section of the UniProt database. Duplicates were removed. The procedure yielded 373 protein sequences that are described in **Supplemental Table S1**. Human proteins are expressed as UniProt entry names, if not discussed in detail.

Proteins from HCMV (human herpesvirus 5, Tax Id: 295027; 168 proteins) were dissected into heptapeptides overlapped by six residues (that is, MPATDTN, PATDTNS, ATDTNST, TDTNSTH, and so forth). Then, each viral heptapeptide served as a probe to screen the library for exact matches within the proteins related to neuronal migration.

The viral heptapeptides shared with the neuronal migration-associated proteins were successively analyzed for occurrences in the entire human proteome using the Peptide Match program (https://research.bioinformatics.udel.edu/peptidematch/index.jsp) (33). The 373 human proteins listed in **Supplemental Table 1** were filtered out.

The Immune Epitope Database (IEDB; www.iedb.org) resource (34) was used to investigate the immunological potential of the peptide sharing among HCMV and human proteins related to neuronal migration. Only epitopes that had been experimentally validated as immunopositive in the human host were considered.

## Results and Discussion

### Heptapeptide Sharing Between HCMV and Human Proteins Related to Neuronal Migration

Following the procedure described under *Methods*, we found that 41 HCMV heptapeptides are repeatedly distributed among 26 proteins associated with neuronal migration (see **Table 1**). An example of potential neuropathological relevance is the protein expression level in the hippocampus, a brain region where neurogenesis occurs in the adult stage.

**Table 1:** Heptapeptide sharing between HCMV and human proteins related to neuronal migration.

HCMV heptapeptide^1^	Occurrences in the human proteome^2^	Occurrences in the set of proteins related to neuronal migration^3^	Human proteins related to neuronal migration^4^ ^,^ ^5^		
			UniProt Name	Cellular location^6^	Protein expression in the hippocampus^7^ ^,^ ^8^
AVENGDS	0	1	SAV1	I	l
DRGGGGG	0	1	SHH	I	–
KPGASAA	0	1	MAGI2	I	M
LKPGASA	0	1	MAGI2	I	M
LLLPPPS	0	1	ACK1	I	M
QTVTSTP	0	2	SMAD2 SMAD3	I I	h m
STTAAAA	0	1	BARH2	I	–
YQRFLRE	0	1	ACK1	I	M
AAGPPEA	1	1	CAC1B	M	l
RRERERR	1	1	CAC1B	M	l
SGLGDLS	1	1	AP2A	I	l
TDSSLEA	1	1	MK10	I	M
PPAPRGP	2	1	RTN4	I	h
SGSSASS	2	1	LMNA	I	h
SSGSSAS	3	1	LMNA	I	h
SAVAAAA	4	1	SOX1	I	–
SEEEDDD	5	1	TOP2B	I	h
SGGAGGG	5	1	SMAD2	I	h
DNLTLWT	6	1	1433E	I	h
LAVADLL	11	2	5HT2B DRD2	M I	nd m
EDDDDDD	21	1	FGFR1	I	M
AAAAASS	24	1	SOX1	I	–
SSGGGGG	26	1	ALK	I	h
EEEDDDD	27	1	APBB1	I	M
AAAAAAP	30	2	CADM1 VAX1	I I	nd –
DDDDDDD	30	1	FGFR1	I	M
QQPPPPP	33	1	BARH2	I	–
GAGGGGG	40	1	SOX1	I	–
AVAAAAA	43	1	SOX1	I	–
EEEEEDD	47	1	APBB1	I	M
VAAAAAA	51	1	SOX1	I	–
AGGGGGG	56	2	ALK SOX1	I I	h –
GGGGGGA	62	2	ALK SOX1	I I	h –
AAAAAAS	70	1	SOX1	I	–
QPPPPPP	70	1	BARH2	I	–
SAAAAAA	72	1	VAX1	I	nd
EEEEEED	140	3	ndF4 PAK3 RTN4	I I I	nd – h
GGGGGGG	170	2	ALK SOX1	I I	h –
SSSSSSS	173	1	ULK1	I	–
AAAAAAA	258	4	BARH2 CADM1 SOX1 VAX1	I I I I	– nd – nd
EEEEEEE	301	4	CELR2 NDF4 PAK3 RTN4	M I I I	M nd – h

1 HCMV heptapeptide sequences in 1-letter aa code.

2 HCMV heptapeptide occurrences in the human proteome, with proteins related to neuronal migration (**Table S1**) filtered out.

3 HCMV heptapeptide occurrences in human proteins related to neuronal migration.

4 Human proteins related to neuronal migration and sharing HCMV heptapeptide(s). Proteins indicated according to UniProtKB entry name.

5 Data from the Human Protein Atlas (35).

6 I, intracellular; M, membrane.

7 Expression level: nd, not detected; l, low; m, medium; h, high.

8 Data pending.

The viral versus human peptide sharing displayed in **Table 1** is specific, unexpected, intensive, and endowed with an immunologic potential, as outlined in the following paragraphs.

Specificity: The shared heptapeptides found in this analysis are, in general, scarcely represented in the entire human proteome assumed as a control (see **Table 1**, 1st column). In other words, most of the matches illustrated in **Table 1** do not reflect an unspecific viral heptapeptide over-representation throughout the human proteome. Extreme examples for the specificity of the heptapeptide overlap are the sequences AVENGDS, DRGGGGG, INKRVKR, KPGASAA, LKPGASA, QTVTSTP, SSSSTSH, and YQRFLRE that are uniquely present in proteins related to neuronal migration and absent in the remaining human proteins (see **Table 1**). Actually, the heptapeptides AVENGDS, DRGGGGG, INKRVKR, KPGASAA, LKPGASA, QTVTSTP, SSSSTSH, and YQRFLRE are HCMV molecular signatures of the human proteins associated with neuronal migration SAV1, SHH, MAGI2, SMAD2, SMAD3, ULK1, and ACK1, respectively. Exceptions to such a specific sharing are represented by simple aa repeats such as EEEEEED. GGGGGGG, SSSSSSS, AAAAAAA, and EEEEEEE, known for being common in eukaryotic proteomes (36, 37).

Unexpectedness: The heptapeptide sharing between HCMV and human proteins associated with neuronal migration is largely unexpected in light of the fact that the probability of finding the same heptapeptide fragment in two proteins is 1 out of 20^7^.

**Figure 1:**
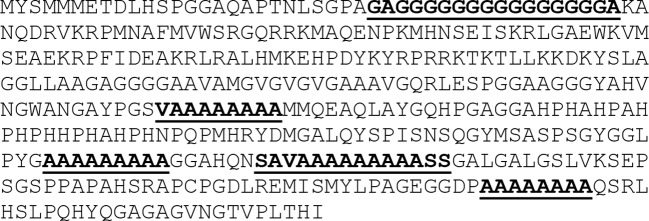
Distribution of overlapping HCMV heptapeptides through SOX1 primary aa sequence. HCMV peptide sequences are highlighted.

Intense peptide sharing: The overlap is not just extensive by affecting many of proteins examined, but also intensive, meaning that, in spite of the low probability, many of the proteins associated with neuronal migration share more than one HCMV heptapeptide. An example is the human transcription factor SOX1 that shares 10 heptapeptides with HCMV (see **Table 1**). Of note, the 10 viral heptapeptide matches that are disseminated along the SOX1 primary amino acid consecutively overlap to form long peptide stretches which may be targeted by anti-HCMV immune responses (see **Figure 1**).

Immunological potential: Finally, many of the heptapeptides shared between HCMV and the 26 human proteins related to neuronal migration are endowed with an immunologic potential by being part of epitopes that have been experimentally validated as immunopositive in humans (see **Table 2**).

**Table 2:** Immunopositive epitopes containing heptapeptides shared between HCMV and human proteins associated with neuronal migration.

IEDB ID^1^	Epitopes^2,3^	IEDB ID^1^	Epitopes^2,3^
71055	vsnappvaspsiLKPGASAA	512030	asggAAAAAAAPaap
424109	AVENGDSgsryyy	515004	epAAAAASSacaapsq
429240	aSAAAAAAAAlly	516191	gAAAAAAAPaapaapr
432006	qtdprAGGGGGGdy	516566	GGGGGGAaaagray
433931	fvrepedEEEEEEEEEED	517250	gptGGGGGGGfntvgr
440752	srevftSSSSSSS	518048	hqpsasggAAAAAAAPa
440782	sSSGGGGGGGrfssssgy	519007	ipSAAAAAAAAgria
441180	tSSSSSSSrqtrpilk	519995	kkwenEEEEEEEEqppp
456753	mAAAAAAAPs	521695	lppkpgtmEEEEEDDdy
457859	QPPPPPPpm	525008	rlAAAAAAAqsvy
465590	glAAGPPEA	525963	sggAAAAAAAPaapa
466037	gprpAAAAAAAtpav	530324	ypdppgtmEEEEEDDd
474480	AAAAAAAqsvy	541856	esnGGGGGGGAgsgggp
483230	qeSAAAAAA	542212	gaavVAAAAAASm
510536	AAAAAAAAPaaaat	542215	GAGGGGGeagagggaaava
510982	AGGGGGGAaaagray	544474	pQPPPPPPp

^1^Epitope IEDB IDs are listed according to numerical order. Further details and references are reported in http://www.iedb.org/.

^2^Epitope peptides are given in one-letter codes.

^3^Epitope fragments shared between HMCV and human proteins associated with neuronal migration are indicated in capital letters.

### Immunological Relevance of the Heptapeptide Sharing Between HCMV and Human Proteins Associated With Neuronal Migration

**Tables 1** and **2** support the possibility that immune responses against HCMV may cross-react with brain proteins involved in neuronal connectivity, synaptogenesis, and transmitter networks. Although the protein cell location is mainly intracellular (see **Table 1**), proteins involved in the viral overlap nonetheless remain fully accessible to immune cross-reactions, given the availability of intracellular antigens to the immune system, which is a well-known phenomenon (38, 39). Immune cross-reactions with these proteins can (1) impair brain development, structure, and function; (2) alter cognitive processes and behavior; and (3) be involved in complex mental disorders: in particular, disorders from the psychotic spectrum.

Indeed, examples are, *inter alia*:

1. BarH-like 2 homeobox protein (BARH2) and sonic hedgehog protein (SHH) contribute to establish the positional identities of progenitor cells in the diencephalon (40), while alterations of BARH2 and SHH can affect cerebellum development (41, 42). Notably, reduced cerebellar volume has been reported in first-time psychotic episodes (43).
2. Ventral anterior homeobox 1 (VAX1) is a transcription factor, and its deficit causes severe depletion of GABAergic neurons in the neocortex (44), thus possibly triggering the emergence of disorders within the psychotic spectrum. Indeed, a deficit in GABAergic system is one of the predominant pathophysiological features in psychotic disorders (45–47).
3. Fibroblast growth factor receptor 1 (FGFR1) may be involved in aberrant dopaminergic firing in psychotic disorders. Altered FGFR1 affects development and function of dopamine neurons, resulting in psychotic disorders in transgenic mice (48).
4. Cell adhesion molecule 1 (CADM1) expression has been detected in glutamatergic neurons, including the granule cells of the dentate gyrus, the pyramidal cells of the CA1 and CA3 regions (namely, in parvalbumin-positive neurons in the CA3 region), and in a subset of GABAergic neurons in the hippocampus (49, 50).
5. The 1433E epsilon protein (1433E or tyrosine 3-monooxygenase/tryptophan 5-monooxygenase activation protein [YWHAE]); 5-hydroxytryptamine receptor 2B (5HT2B or serotonin receptor 2B); and dopamine D2 receptor (DRD2) are three proteins, that—when altered—appear to be involved in the genesis of psychotic disorders. Actually, theories on potential causes of psychotic disorders assign a causal role to altered serotonin and dopamine neurotransmission (51–58). Specifically, the HCMV peptide sharing with 5HT2B and DRD2 consists of the heptapeptide LAVADLL (**Table 1**). The HCMV LAVADLL peptide is present in the transmembrane domain 2 (TMD2) of 5-HT2B and is involved in the interaction with TMD7 that allows the human 5-HT2B to adopt a conformation able to bind the neurotransmitter serotonine (59). Moreover, the LAVADLL sequence is endowed with an immunogenic potential by being part of the epitope KLAVADLEK (IEDB ID: 213202), derived from human centromere protein F (aa pos 557–565) (60). Therefore, cross-reactions targeting LAVADLL may hit multiple proteins involved in neurotransmission as well as centriolar proteins involved in brain malformations (microcephaly and ocular anomalies) (61).
6. The transcription factor Sex-determining Region Y-related HMG-box 1 (SOX1) is uniquely expressed at a high level in the majority of telencephalic neurons that constitute the ventral striatum (62), a brain area closely associated with decision making and belonging to the reward-salience circuitry (i.e., ventral striatum, dorsal caudate, and anterior cingulate cortex) (63–65). SOX1 regulates the neural primordia and promotes neurogenesis not only by acting as a transcription factor but also by forming protein-protein interactions through its COOH-terminus (66). Of note, the HCMV *versus* SOX1 peptide overlap is mainly allocated in the COOH-terminus (**Figure 1**). Consequently, cross-reactions targeting the SOX1 C-terminus may have multiple pathologic consequences, from disruption of the molecular network underlying neurodevelopment to alteration of specific neural circuits that produce complex behavior.
7. The anaplastic lymphoma kinase (ALK) protein is a tyrosine kinase receptor that, when altered, is involved in psychotic disorders (67, 68) and in neuroblastoma, a common neoplasm of early childhood that arises from cells of the primitive neural crest, giving rise to the adrenal medulla and the sympathetic nervous system (69).
8. The serine/threonine-protein kinase (PAK3) (also known as oligophrenin-3) plays a role in dendrite spine morphogenesis as well as synapse formation and plasticity, and its dysregulation may lead to synaptic deficits in psychotic disorders (70–72).
9. The reticulon-4 (RTN4) protein is implicated in the stabilization of wiring and restriction of plasticity in the adult central nervous system (73). RTN4 is differentially expressed in the dorsolateral prefrontal cortex from individuals with psychotic disorders (74).
10. MAGI2 is a membrane-associated guanylate kinase that acts as a scaffold molecule at synaptic junctions by assembling neurotransmitter receptors and cell adhesion proteins. MAGI2 seems to be involved in psychotic disorders (75–77).
11. The brain calcium channel III or voltage-dependent N-type calcium channel subunit alpha-1B (CAC1B) may have a key role in etiology of bipolar disorder and psychosis (78).

The variety of proteins involved in peptide sharing with HCMV presented here is consistent with the complex multifactorial nature of mental disorders in general, and psychosis in particular. These proteins were examined in the present study in light of their involvement with neuronal migration, while it is highly likely that any alteration of their function or structure may affect higher cognitive processes through impairment of different mechanisms above and beyond migration (i.e., axon guidance, neurotransmission, excitatory-inhibitory balance, oscillatory neuronal firing, and others). Notably, these mechanisms can also be directly affected by cross-reactive targeting of proteins allowing membrane excitability (26–30), in a complex endotypical scenario that mirrors the phenotypical complexity of mental disorders without the need for a biunivocal match between them.

## Conclusions

The present study demonstrates that numerous human proteins related to neuronal migration are involved in a specific heptapeptide overlap with HCMV. Such a wide peptide sharing supports the possibility that, following HCMV active infection, anti-HCMV human immune responses may cross-react with proteins involved in peptide sharing with the herpesvirus. In the case of cross-reactions, neuropathological consequences might include the development of mental disorders, such as psychotic syndromes. In fact, the 26 human proteins listed in **Table 1** hold the key to specifying brain processes, such as neuronal connectivity, synaptogenesis, and neurotransmission in a prolonged temporal window that runs from fetal-early postnatal neurodevelopment to adult neurogenesis. In the context of peptide sharing described here, GABAergic and glutamatergic circuitry might play a central role, with disturbances potentially leading to psychotic syndromes by altering excitatory-inhibitory balance in oscillating brain networks underpinning higher cognitive functions (79–84). Different strategies could allow to test this hypothesis *in vivo*. Observationally, sera from human patients suffering from psychotic disorders might be examined for immunoreactivity against the sequences analyzed here. Causally, animal models of neuropsychiatric disorders might be obtained by immunizing pregnant and young animals with the same sequences.

## Data Availability Statement

All datasets analyzed for this study were retrieved from publicly accessible curated databases: UniProtKB (http://www.uniprot.org/), The Immune Epitope Database (IEDB; http://www.iedb.org/), and the Human Protein Atlas (https://www.proteinatlas.org/).

## Author Contributions

GL formulated the hypothesis, analyzed the data, and wrote the manuscript. GL, AF, and BS interpreted the data and revised and finalized the manuscript.

## Funding

We acknowledge support for the Article Processing Charge from the DFG (German Research Foundation, 393148499) and the Open Access Publication Fund of the University of Greifswald. GL is supported by a “Gerhard Domagk” research grant awarded by the University of Greifswald Medical School.

## Conflict of Interest

AF received consulting fees from Bayer and Novartis and honoraria for oral presentations from Novartis, Böhringer-Ingelheim, Lilly, and Biogen Idec.

The remaining authors declare that the research was conducted in the absence of any commercial or financial relationships that could be construed as a potential conflict of interest.

## References

[1] Suter TACS Jaworski A Cell migration and axon guidance at the border between central and peripheral nervous system. Science. (2019) 365(6456):eaaw8231. 10.1126/science.aaw8231 31467195 PMC6733266

[2] Ohtaka-Maruyama C Okado H Molecular Pathways Underlying Projection Neuron Production and Migration during Cerebral Cortical Development. Front Neurosci (2015) 9:447. 10.3389/fnins.2015.00447 26733777 PMC4682034

[3] Ayala R Shu T Tsai LH Trekking across the brain: the journey of neuronal migration. Cell (2007) 128:29–43. 10.1016/j.cell.2006.12.021 17218253

[4] Falnikar A Tole S Liu M Liu JS Baas PW Polarity in migrating neurons is related to a mechanism analogous to cytokinesis. Curr Biol (2013) 23:1215–20. 10.1016/j.cub.2013.05.027 PMC3710514 23791725

[5] Falnikar A Baas PW Neuronal migration re-purposes mechanisms of cytokinesis. Cell Cycle (2013) 12:3577–8. 10.4161/cc.26821 PMC3903703 24131923

[6] Saghatelyan A Role of blood vessels in the neuronal migration. Semin Cell Dev Biol (2009) 20:744–50. 10.1016/j.semcdb.2009.04.006 19374951

[7] Spalice A Parisi P Nicita F Pizzardi G Del Balzo F Iannetti P Neuronal migration disorders: clinical, neuroradiologic and genetics aspects. Acta Paediatr (2009) 98:421–33. 10.1111/j.1651-2227.2008.01160.x 19120042

[8] Valiente M Marín O Neuronal migration mechanisms in development and disease. Curr Opin Neurobiol (2010) 20:68–78. 10.1016/j.conb.2009.12.003 20053546

[9] Muraki K Tanigaki K Neuronal migration abnormalities and its possible implications for schizophrenia. Front Neurosci (2015) 9:74. 10.3389/fnins.2015.00074 25805966 PMC4354421

[10] Fukuda T Yanagi S Psychiatric behaviors associated with cytoskeletal defects in radial neuronal migration. Cell Mol Life Sci (2017) 74(19):3533–52. 10.1007/s00018-017-2539-4 PMC11107632 28516224

[11] Jakob H Beckmann H Prenatal developmental disturbances in the limbic allocortex in schizophrenics. J Neural Transm (1986) 65:303–26. 10.1007/bf01249090 3711886

[12] Krimer LS Herman MM Saunders RC Boyd JC Hyde TM Carter JM A qualitative and quantitative analysis of the entorhinal cortex in schizophrenia. Cereb Cortex (1997) 7:732–9. 10.1093/cercor/7.8.732 9408037

[13] Weinberger DR The neurodevelopmental origins of schizophrenia in the penumbra of genomic medicine. World Psychiatry (2017) 16(3):225–6. 10.1002/wps.20474 PMC5608828 28941096

[14] Di Cristo G Development of cortical GABAergic circuits and its implications for neurodevelopmental disorders. Clin Genet (2007) 72:1–8. 10.1111/j.1399-0004.2007.00822.x 17594392

[15] Crandall JE McCarthy DM Araki KY Sims JR Ren JQ Bhide PG Dopamine 660 receptor activation modulates GABA neuron migration from the basal forebrain to the cerebral cortex. J Neurosci (2007) 27:3813–22. 10.1523/JNEUROSCI.5124-06.2007 PMC2711976 17409246

[16] Li J-T Su Y-A Wang H-L Zhao Y-Y Liao X-M Wang X-D Repeated Blockade of NMDA Receptors During 662 Adolescence Impairs Reversal Learning and Disrupts GABAergic 663 Interneurons in Rat Medial Prefrontal Cortex. Front Mol Neurosci (2016) 9:17. 10.3389/fnmol.2016.00017 26973457 PMC4776083

[17] Shinmura Y Kosugi I Aiba-Masago S Baba S Yong LR Tsutsui Y Disordered migration and loss of virus-infected neuronal cells in developing mouse brains infected with murine cytomegalovirus. Acta Neuropathol (1997) 93:551–7. 10.1007/s004010050651 9194893

[18] Han D Byun SH Kim J Kwon M Pleasure SJ Ahn JH Human Cytomegalovirus IE2 protein disturbs brain development by the dysregulation of neural stem cell maintenance and the polarization of migrating neurons. J Virol (2017) 91(17):pii: e00799–17. 10.1128/JVI.00799-17 PMC5553173 28615204

[19] Yoon JY Danielson B Mathis D Karamchandani J Munoz DG Cytomegalovirus in the human dentate gyrus and its impact on neural progenitor cells: report of two cases. Clin Neuropathol (2017) 36(5):240–5. 10.5414/NP301020 28502321

[20] Blomström A Karlsson H Wicks S Yang S Yolken RH Dalman C Maternal antibodies to infectious agents and risk for non-affective psychoses in the offspring-a matched case-control study. Schizophr Res (2012) 140:25–30. 10.1016/j.schres.2012.06.035 22819777

[21] Dalman C Allebeck P Gunnell D Harrison G Kristensson K Lewis G Infections in the CNS during childhood and the risk of subsequent psychotic illness: a cohort study of more than one million Swedish subjects. Am J Psychiatry (2008) 165(1):59–65. 10.1176/appi.ajp.2007.07050740 18056223

[22] Dickerson F Kirkpatrick B Boronow J Stallings C Origoni A Yolken R Deficit schizophrenia: association with serum antibodies to cytomegalovirus. Schizophr Bull (2006) 32(2):396–400. 10.1093/schbul/sbi054 16166610 PMC2632221

[23] Shirts BH Prasad KM Pogue-Geile MF Dickerson F Yolken RH Nimgaonkar VL Antibodies to cytomegalovirus and Herpes Simplex Virus 1 associated with cognitive function in schizophrenia. Schizophr Res (2008) 106:268–74. 10.1016/j.schres.2008.07.017 PMC2615667 18801645

[24] Krause DL Weidinger E Matz J Wildenauer A Wagner JK Obermeier M Infectious agents are associated with psychiatric diseases. Ment Illn. (2012) 4:e10. 10.4081/mi.2012.e10 25478103 PMC4253361

[25] Houenou J d’Albis MA Daban C Hamdani N Delavest M Lepine JP Cytomegalovirus seropositivity and serointensity are associated with hippocampal volume and verbal memory in schizophrenia and bipolar disorder. Prog Neuropsychopharmacol Biol Psychiatry (2014) 48:142–8. 10.1016/j.pnpbp.2013.09.003 24083998

[26] Lucchese G Capone G Kanduc D Peptide sharing between influenza A H1N1 hemagglutinin and human axon guidance proteins. Schizophr Bull (2014) 40:362–75. 10.1093/schbul/sbs197 PMC3932078 23378012

[27] Lucchese G Confronting JC virus and Homo sapiens biological signatures. Front Biosci (2013) 18:716–24. 10.2741/4133 23276955

[28] Lucchese G Understanding neuropsychiatric diseases, analyzing the peptide sharing between infectious agents and the language-associated NMDA 2A protein. Front Psychiatry (2016) 7:60. 10.3389/fpsyt.2016.00060 27148089 PMC4827103

[29] Lucchese G From toxoplasmosis to schizophrenia *via* NMDA dysfunction: peptide overlap between Toxoplasma gondii and N-Methyl-d-Aspartate Receptors as a potential mechanistic link. Front Psychiatry (2017) 8:37. 10.3389/fpsyt.2017.00037 28360866 PMC5350139

[30] Lucchese G Stahl B Peptide sharing between viruses and DLX proteins: A potential cross-reactivity pathway to neuropsychiatric disorders. Front Neurosci (2018) 12:150. 10.3389/fnins.2018.00150 29618965 PMC5871705

[31] Lucchese G Flöel A Stahl B Cross-reactivity as a mechanism linking infections to stroke. Front Neurol (2019) 10:469. 10.3389/fneur.2019.00469 31156531 PMC6528689

[32] Magrane M UniProt Consortium. UniProt Knowledgebase: a hub of integrated protein data. Database (Oxford) (2011) 2011:bar009. 10.1093/database/bar009 21447597 PMC3070428

[33] Chen C Li Z Huang H Suzek BE Wu CH UniProt Consortium. A fast Peptide Match service for UniProt Knowledgebase. Bioinformatics (2013) 29:2808–9. 10.1093/bioinformatics/btt484 PMC3799477 23958731

[34] Vita R Overton JA Greenbaum JA Ponomarenko J Clark JD Cantrell JR The immune epitope database 2.0. Nucleic Acids Res (2010) 38:D854–862. 10.1093/nar/gkp1004 PMC2808938 19906713

[35] Uhlen M A pathology atlas of the human cancer transcriptome. Science (2017). 10.1126/science.aan2507 28818916

[36] Karlin S Brocchieri L Bergman A Mrazek J Gentles AJ Amino acid runs in eukaryotic proteomes and disease associations. Proc Natl Acad Sci U S A. (2002) 99:333–8. 10.1073/pnas.012608599 PMC117561 11782551

[37] Lucchese G Kanduc D Single amino acid repeats connect viruses to neurodegeneration. Curr Drug Discovery Technol (2014) 11:214–9. 10.2174/1570163811666140212112300 24521198

[38] Greenlee JE Clawson SA Hill KE Wood B Clardy SL Tsunoda I Anti-Yo Antibody Uptake and Interaction with Its Intracellular Target Antigen Causes Purkinje Cell Death in Rat Cerebellar Slice Cultures: A Possible Mechanism for Paraneoplastic Cerebellar Degeneration in Humans with Gynecological or Breast Cancers. PloS One (2015) 10:e0123446. 10.1371/journal.pone.0123446 25885452 PMC4401511

[39] Racanelli V Prete M Musaraj G Dammacco F Perosa F Autoantibodies to intracellular antigens: generation and pathogenetic role. Autoimmun Rev (2011) 10:503–8. 10.1016/j.autrev.2011.03.001 21397735

[40] Ding Q Balasubramanian R Zheng D Liang G Gan L Barhl2 Determines the Early Patterning of the Diencephalon by Regulating Shh. Mol Neurobiol (2017) 54(6):4414–20. 10.1007/s12035-016-0001-5 PMC5191999 27349434

[41] Bulfone A Menguzzato E Broccoli V Marchitiello A Ga ttuso C Mariani M Barhl1, a gene belonging to a new subfamily of mammalian homeobox genes, is expressed in migrating neurons of the CNS. Hum Mol Genet (2000) 9(9):1443–52. 10.1093/hmg/9.9.1443 10814725

[42] De Luca A Cerrato V Fucà E Parmigiani E Buffo A Leto K Sonic hedgehog patterning during cerebellar development. Cell Mol Life Sci (2016) 73:291–303. 10.1093/hmg/9.9.1443 26499980 PMC11108499

[43] Bottmer C Bachmann S Pantel J Essig M Amann M Schad LR Reduced cerebellar volume and neurological soft signs in first-episode schizophrenia. Psychiatry Res (2005) 140:239–50. 10.1016/j.pscychresns.2005.02.011 16288852

[44] Taglialatela P Soria JM Caironi V Moiana A Bertuzzi S Compromised generation of GABAergic interneurons in the brains of Vax1-/- mice. Development (2004) 131:4239–49. 10.1242/dev.01299 15280216

[45] Costa E Davis JM Dong E Grayson DR Guidotti A Tremolizzo L Veldic M. A GABAergic cortical deficit dominates schizophrenia pathophysiology. Crit Rev Neurobiol (2004) 16:1–23. 10.1615/critrevneurobiol.v16.i12.10 15581395

[46] Skilbeck KJ Johnston GAR Hinton T Long-lasting effects of early-life intervention in mice on adulthood behaviour, GABA(A) receptor subunit expression and synaptic clustering. Pharmacol Res (2018) 128:179–189. 10.1016/j.phrs.2017.09.021 28970177

[47] Tse MT Piantadosi PT Floresco SB Prefrontal cortical gamma-aminobutyric acid transmission and cognitive function: drawing links to schizophrenia from preclinical research. Biol Psychiatry (2015) 77(11):929–39. 10.1016/j.biopsych.2014.09.007 25442792

[48] Klejbor I Myers JM Hausknecht K Corso TD Gambino AS Morys J Fibroblast growth factor receptor signaling affects development and function of dopamine neurons-inhibition results in a schizophrenia-like syndrome in transgenic mice. J Neurochem (2006) 97:1243–58. 10.1111/j.1471-4159.2006.03754.x 16524369

[49] Thomas LA Akins MR Biederer T Expression and adhesion profiles of SynCAM molecules indicate distinct neuronal functions. J Comp Neurol (2008) 510:47–67. 10.1002/cne.21773 18615557 PMC2543944

[50] Yamada A Inoue E Deguchi-Tawarada M Matsui C Togawa A Nakatani T Necl-2/CADM1 interacts with ErbB4 and regulates its activity in GABAergic neurons. Mol Cell Neurosci (2013) 56:234–43. 10.1016/j.mcn.2013.06.003 23769722

[51] Cheah PS Ramshaw HS Thomas PQ Toyo-Oka K Xu X Martin S Neurodevelopmental and neuropsychiatric behaviour defects arise from 14-3-3ζ deficiency. Mol Psychiatry (2012) 17:451–66. 10.1038/mp.2011.158 22124272

[52] Kuroki T Nagao N Nakahara T Neuropharmacology of second-generation antipsychotic drugs: a validity of the serotonin-dopamine hypothesis. Prog Brain Res (2008) 172:199–212. 10.1016/S0079-6123(08)00910-2 18772034

[53] Remington G Alterations of dopamine and serotonin transmission in schizophrenia. Prog Brain Res (2008) 172:117–40. 10.1016/S0079-6123(08)00906-0 18772030

[54] Murray RM Lappin J Di Forti M Schizophrenia: from developmental deviance to dopamine dysregulation. Eur Neuropsychopharmacol (2008) 18(Suppl 3):S129–34. 10.1016/j.euroneuro.2008.04.002 18499406

[55] Inta D Meyer-Lindenberg A Gass P Alterations in postnatal neurogenesis and dopamine dysregulation in schizophrenia: a hypothesis. Schizophr Bull (2011) 37(4):674–80. 10.1093/schbul/sbq134 PMC3122276 21097511

[56] Howes OD Nour MM Dopamine and the aberrant salience hypothesis of schizophrenia. World Psychiatry (2016) 15:3–4. 10.1002/wps.20276 26833595 PMC4780291

[57] Lau CI Wang HC Hsu JL Liu ME Does the dopamine hypothesis explain schizophrenia? Rev Neurosci (2013) 24:389–400. 10.1515/revneuro-2013-0011 23843581

[58] Heinz A Schlagenhauf F Dopaminergic dysfunction in schizophrenia: salience attribution revisited. Schizophr Bull (2010) 36:472–85. 10.1093/schbul/sbq031 PMC2879696 20453041

[59] Manivet P Schneider B Smith JC Choi DS Maroteaux L Kellermann O The serotonin binding site of human and murine 5-HT2B receptors: molecular modeling and site-directed mutagenesis. J Biol Chem (2002) 277:17170–8. 10.1074/jbc.M200195200 11859080

[60] Granados DP Sriranganadane D Daouda T Zieger A Laumont CM Caron-Lizotte O Impact of genomic polymorphisms on the repertoire of human MHC class I-associated peptides. Nat Commun (2014) 5:3600. 10.1038/ncomms4600 24714562 PMC3996541

[61] Waters AM Asfahani R Carroll P Bicknell L Lescai F Bright A The kinetochore protein, CENPF, is mutated in human ciliopathy and microcephaly phenotypes. J Med Genet (2015) 52:147–56. 10.1136/jmedgenet-2014-102691 PMC4345935 25564561

[62] Ekonomou A Kazanis I Malas S Wood H Alifragis P Denaxa M Neuronal migration and ventral subtype identity in the telencephalon depend on SOX1. PloS Biol (2005) 3:e186. 10.1371/journal.pbio.0030186 15882093 PMC1110909

[63] Schmidt A Antoniades M Allen P Egerton A Chaddock CA Borgwardt S Longitudinal alterations in motivational salience processing in ultra-high-risk subjects for psychosis. Psychol Med (2016) 4:1–12. 10.1017/S0033291716002439 PMC5216461 27697078

[64] Stegmayer K Horn H Federspiel A Razavi N Bracht T Laimböck K Ventral striatum gray matter density reduction in patients with schizophrenia and psychotic emotional dysregulation. NeuroImage Clin (2013) 4:232–9. 10.1016/j.nicl.2013.12.007 PMC3895617 24455473

[65] Esslinger C Englisch S Inta D Rausch F Schirmbeck F Mier D Ventral striatal activation during attribution of stimulus saliency and reward anticipation is correlated in unmedicated first episode schizophrenia patients. Schizophr Res (2012) 140:114–21. 10.1016/j.schres.2012.06.025 22784688

[66] Kan L Israsena N Zhang Z Hu M Zhao LR Jalali A Sox1 acts through multiple independent pathways to promote neurogenesis. Dev Biol (2004) 269:580–94. 10.1016/j.ydbio.2004.02.005 15110721

[67] Kunugi H Hashimoto R Okada T Hori H Nakabayashi T Baba A Possible association between nonsynonymous polymorphisms of the anaplastic lymphoma kinase (ALK) gene and schizophrenia in a Japanese population. J Neural Transm (Vienna) (2006) 113:1569–73. 10.1007/s00702-006-0436-3 16604305

[68] Bilsland JG Wheeldon A Mead A Znamenskiy P Almond S Waters KA Behavioral and neurochemical alterations in mice deficient in anaplastic lymphoma kinase suggest therapeutic potential for psychiatric indications. Neuropsychopharmacology (2008) 33:685–700. 10.1038/sj.npp.1301446 17487225

[69] Mossé YP Laudenslager M Longo L Cole KA Wood A Attiyeh EF Identification of ALK as a major familial neuroblastoma predisposition gene. Nature. (2008) 455:930–5. 10.1038/nature07261 PMC2672043 18724359

[70] Datta D Arion D Corradi JP Lewis DA Altered expression of CDC42 signaling pathway components in cortical layer 3 pyramidal cells in schizophrenia. Biol Psychiatry (2015) 78:775–85. 10.1016/j.biopsych.2015.03.030 PMC4600637 25981171

[71] Morrow EM Kane A Goff DC Walsh CA Sequence analysis of P21-activated kinase 3 (PAK3) in chronic schizophrenia with cognitive impairment. Schizophr Res (2008) 106:265–7. 10.1016/j.schres.2008.08.021 PMC2631562 18805672

[72] Kim MJ Biag J Fass DM Lewis MC Zhang Q Fleishman M Functional analysis of rare variants found in schizophrenia implicates a critical role for GIT1-PAK3 signaling in neuroplasticity. Mol Psychiatry (2017) 22(3):417–29. 10.1038/mp.2016.98 PMC6186433 27457813

[73] Schwab ME Functions of Nogo proteins and their receptors in the nervous system. Nat Rev Neurosci (2010) 11:799–811. 10.1038/nrn2936 21045861

[74] Martins-de-Souza D Gattaz WF Schmitt A Rewerts C Maccarrone G Dias-Neto E Prefrontal cortex shotgun proteome analysis reveals altered calcium homeostasis and immune system imbalance in schizophrenia. Eur Arch Psychiatry Clin Neurosci (2009) 259:151–63. 10.1007/s00406-008-0847-2 19165527

[75] Koide T Banno M Aleksic B Yamashita S Kikuchi T Kohmura K Common variants in MAGI2 gene are associated with increased risk for cognitive impairment in schizophrenic patients. PloS One (2012) 7:e36836. 10.1371/journal.pone.0036836 22649501 PMC3359314

[76] Potkin SG Turner JA Guffanti G Lakatos A Fallon JH Nguyen DD A genome-wide association study of schizophrenia using brain activation as a quantitative phenotype. Schizophr Bull (2009) 35:96–108. 10.1093/schbul/sbn155 19023125 PMC2643953

[77] Karlsson R Graae L Lekman M Wang D Favis R Axelsson T MAGI1 copy number variation in bipolar affective disorder and schizophrenia. Biol Psychiatry (2012) 71:922–30. 10.1016/j.biopsych.2012.01.020 22381734

[78] Curtis D Vine AE McQuillin A Bass NJ Pereira A Kandaswamy R Case-case genome-wide association analysis shows markers differentially associated with schizophrenia and bipolar disorder and implicates calcium channel genes. Psychiatr Genet (2011) 21:1–4. 10.1097/YPG.0b013e3283413382 21057379 PMC3024533

[79] Grent-’t-Jong T Rivolta D Sauer A Grube M Singer W Wibral M MEG-measured visually induced gamma-band oscillations in chronic schizophrenia: Evidence for impaired generation of rhythmic activity in ventral stream regions. Schizophr Res (2016) 176:177–85. 10.1016/j.schres.2016.06.003 27349815

[80] McNally JM McCarley RW Gamma band oscillations: a key to understanding schizophrenia symptoms and neural circuit abnormalities. Curr Opin Psychiatry (2016) 29:202–10. 10.1097/YCO.0000000000000244 PMC4901383 26900672

[81] Heckers S Konradi C GABAergic mechanisms of hippocampal hyperactivity in schizophrenia. Schizophr Res (2015) 167:4–11. 10.1016/j.schres.2014.09.041 25449711 PMC4402105

[82] Konradi C Yang CK Zimmerman EI Lohmann KM Gresch P Pantazopoulos H Hippocampal interneurons are abnormal in schizophrenia. Schizophr Res (2011) 131:165–173. 10.1016/j.schres.2011.06.007 21745723 PMC3159834

[83] Sun Y Farzan F Barr MS Kirihara K Fitzgerald PB Light GA γ oscillations in schizophrenia: mechanisms and clinical significance. Brain Res (2011) 1413:98–114. 10.1016/j.brainres.2011.06.065 21840506

[84] McNally JM McCarley RW Brown RE Impaired GABAergic neurotransmission in schizophrenia underlies impairments in cortical gamma band oscillations. Curr Psychiatry Rep (2013) 15:346. 10.1007/s11920-012-0346-z 23400808 PMC3595504

