# Supplementary material for: A Peptide Link Between Human Cytomegalovirus Infection, Neuronal Migration, and Psychosis
Source: Front Psychiatry. 2020 May 8;11:349. doi: 10.3389/fpsyt.2020.00349 (PMC7225321; doi:10.3389/fpsyt.2020.00349)
Supplement: Supplementary file 1 [file Table_1.docx]

**Supplemental Table 1.** List of human proteins (373) involved in neuronal migration and analysed for sequence identity to HCMV. Proteins retrieved from UniProt database (www.uniprot.org); described by UniProtKB/Swiss-Prot entry name and number, and listed according to the aa length

| Entry name | Entry number | Protein names | Length |
| --- | --- | --- | --- |
| NREP | Q16612 | Neuronal regeneration-related protein (Neuronal protein 3.1) (Protein p311) | 68 |
| CCL3 | P10147 | C-C motif chemokine 3 (G0/G1 switch regulatory protein 19-1) (Macrophage inflammatory protein 1-alpha) (MIP-1-alpha) (PAT 464.1) (SIS-beta) (Tonsillar lymphocyte LD78 alpha protein) [Cleaved into: MIP-1-alpha(4-69) (LD78-alpha(4-69))] | 92 |
| SDF1 | P48061 | Stromal cell-derived factor 1 (SDF-1) (hSDF-1) (C-X-C motif chemokine 12) (Intercrine reduced in hepatomas) (IRH) (hIRH) (Pre-B cell growth-stimulating factor) (PBSF) [Cleaved into: SDF-1-beta(3-72) SDF-1-alpha(3-67)] | 93 |
| CCL2 | P13500 | C-C motif chemokine 2 (HC11) (Monocyte chemoattractant protein 1) (Monocyte chemotactic and activating factor) (MCAF) (Monocyte chemotactic protein 1) (MCP-1) (Monocyte secretory protein JE) (Small-inducible cytokine A2) | 99 |
| CCKN | P06307 | Cholecystokinin (CCK) [Cleaved into: CCK -58 (CCK58) CCK-58 desnonopeptide ((1-49)-CCK58) CCK-39 (CCK39) CCK -33(CCK33) CCK -25(CCK25) CCK -18(CCK18) CCK -12 (CCK12) CCK -8(CCK8) CCK -7 (CCK7)CCK -5 (CCK5)] | 115 |
| SMS | P61278 | Somatostatin (Growth hormone release-inhibiting factor) [Cleaved into: Somatostatin-28 Somatostatin-14] | 116 |
| TKN1 | P20366 | Protachykinin-1 (PPT) [Cleaved into: Substance P Neurokinin A (NKA) (Neuromedin L) (Substance K) Neuropeptide K (NPK) Neuropeptide gamma C-terminal-flanking peptide] | 129 |
| FABP7 | O15540 | Fatty acid-binding protein, brain (Brain lipid-binding protein) (BLBP) (Brain-type fatty acid-binding protein) (B-FABP) (Fatty acid-binding protein 7) (Mammary-derived growth inhibitor related) | 132 |
| KISS1 | Q15726 | Metastasis-suppressor KiSS-1 (Kisspeptin-1) [Cleaved into: Metastin (KiSS -54) KiSS -14 KiSS -13 KiSS -10] | 138 |
| ANGI | P03950 | Angiogenin (EC 3.1.27.-) (Ribonuclease 5) (RNase 5) | 147 |
| CEND | Q8N111 | Cell cycle exit and neuronal differentiation protein 1 (BM88 antigen) | 149 |
| STMN2 | Q93045 | Stathmin-2 (Superior cervical ganglion-10 protein) (Protein SCG10) | 179 |
| RAP2A | P10114 | Ras-related protein Rap-2a (RbBP-30) | 183 |
| RAP1A | P62834 | Ras-related protein Rap-1A (C21KG) (G-22K) (GTP-binding protein smg p21A) (Ras-related protein Krev-1) | 184 |
| APOD | P05090 | Apolipoprotein D (Apo-D) (ApoD) | 189 |
| RASH | P01112 | GTPase HRas (H-Ras-1) (Ha-Ras) (Transforming protein p21) (c-H-ras) (p21ras) [Cleaved into: GTPase HRas, N-terminally processed] | 189 |
| RASK | P01116 | GTPase KRas (K-Ras 2) (Ki-Ras) (c-K-ras) (c-Ki-ras) [Cleaved into: GTPase KRas, N-terminally processed] | 189 |
| RASN | P01111 | GTPase NRas (Transforming protein N-Ras) | 189 |
| CDC42 | P60953 | Cell division control protein 42 homolog (G25K GTP-binding protein) | 191 |
| BAX | Q07812 | Apoptosis regulator BAX (Bcl-2-like protein 4) (Bcl2-L-4) | 192 |
| RAC1 | P63000 | Ras-related C3 botulinum toxin substrate 1 (Cell migration-inducing gene 5 protein) (Ras-like protein TC25) (p21-Rac1) | 192 |
| RHOA | P61586 | Transforming protein RhoA (Rho cDNA clone 12) (h12) | 193 |
| MRP | P49006 | MARCKS-related protein (MARCKS-like protein 1) (Macrophage myristoylated alanine-rich C kinase substrate) (Mac-MARCKS) (MacMARCKS) | 195 |
| NRTN | Q99748 | Neurturin | 197 |
| EFNA4 | P52798 | Ephrin-A4 (EPH-related receptor tyrosine kinase ligand 4) (LERK-4) | 201 |
| TWST1 | Q15672 | Twist-related protein 1 (Class A basic helix-loop-helix protein 38) (bHLHa38) (H-twist) | 202 |
| GPX1 | P07203 | Glutathione peroxidase 1 (GPx-1) (GSHPx-1) (Cellular glutathione peroxidase) | 203 |
| EFNA1 | P20827 | Ephrin-A1 (EPH-related receptor tyrosine kinase ligand 1) (LERK-1) (Immediate early response protein B61) (Tumor necrosis factor alpha-induced protein 4) (TNF alpha-induced protein 4) [Cleaved into: Ephrin-A1, secreted form] | 205 |
| HSPB1 | P04792 | Heat shock protein beta-1 (HspB1) (28 kDa heat shock protein) (Estrogen-regulated 24 kDa protein) (Heat shock 27 kDa protein) (HSP 27) (Stress-responsive protein 27) (SRP27) | 205 |
| RAB7A | P51149 | Ras-related protein Rab-7a | 207 |
| FGF9 | P31371 | Fibroblast growth factor 9 (FGF-9) (Glia-activating factor) (GAF) (Heparin-binding growth factor 9) (HBGF-9) | 208 |
| GDNF | P39905 | Glial cell line-derived neurotrophic factor (hGDNF) (Astrocyte-derived trophic factor) (ATF) | 211 |
| RAB17 | Q9H0T7 | Ras-related protein Rab-17 | 212 |
| EFNA2 | O43921 | Ephrin-A2 (EPH-related receptor tyrosine kinase ligand 6) (LERK-6) (HEK7 ligand) (HEK7-L) | 213 |
| RAN | P62826 | GTP-binding nuclear protein Ran (Androgen receptor-associated protein 24) (GTPase Ran) (Ras-like protein TC4) (Ras-related nuclear protein) | 216 |
| GRB2 | P62993 | Growth factor receptor-bound protein 2 (Adapter protein GRB2) (Protein Ash) (SH2/SH3 adapter GRB2) | 217 |
| HAND2 | P61296 | Heart- and neural crest derivatives-expressed protein 2 (Class A basic helix-loop-helix protein 26) (bHLHa26) (Deciduum, heart, autonomic nervous system and neural crest derivatives-expressed protein 2) (dHAND) | 217 |
| RRAS | P10301 | Ras-related protein R-Ras (p23) | 218 |
| SCN1B | Q07699 | Sodium channel subunit beta-1 | 218 |
| ARTN | Q5T4W7 | Artemin (Enovin) (Neublastin) | 220 |
| EFNA5 | P52803 | Ephrin-A5 (AL-1) (EPH-related receptor tyrosine kinase ligand 7) (LERK-7) | 228 |
| CD302 | Q8IX05 | CD302 antigen (C-type lectin BIMLEC) (C-type lectin domain family 13 member A) (DEC205-associated C-type lectin 1) (Type I transmembrane C-type lectin receptor DCL-1) (CD antigen CD302) | 232 |
| DDIT4 | Q9NX09 | DNA damage-inducible transcript 4 protein (HIF-1 responsive protein RTP801) (Protein regulated in development and DNA damage response 1) (REDD-1) | 232 |
| NOGG | Q13253 | Noggin | 232 |
| VEGFA | P1592 | Vascular endothelial growth factor A (VEGF-A) (Vascular permeability factor) (VPF) | 232 |
| FGF8 | P55075 | Fibroblast growth factor 8 (FGF-8) (Androgen-induced growth factor) (AIGF) (Heparin-binding growth factor 8) (HBGF-8) | 233 |
| TNFA | P01375 | Tumor necrosis factor (Cachectin) (TNF-alpha) (Tumor necrosis factor ligand superfamily member 2) (TNF-a) [Cleaved into: Tumor necrosis factor, membrane form (N-terminal fragment) (NTF) Intracellular domain 1 (ICD1) Intracellular domain 2 (ICD2) C-domain 1 C-domain 2 Tumor necrosis factor, soluble form] | 233 |
| ASCL1 | P50553 | Achaete-scute homolog 1 (ASH-1) (hASH1) (Class A basic helix-loop-helix protein 46) (bHLHa46) | 236 |
| EDN3 | P14138 | Endothelin-3 (ET-3) (Preproendothelin-3) (PPET3) | 238 |
| EFNA3 | P52797 | Ephrin-A3 (EFL-2) (EHK1 ligand) (EHK1-L) (EPH-related receptor tyrosine kinase ligand 3) (LERK-3) | 238 |
| BCL2 | P10415 | Apoptosis regulator Bcl-2 | 239 |
| FGF13 | Q92913 | Fibroblast growth factor 13 (FGF-13) (Fibroblast growth factor homologous factor 2) (FHF-2) | 245 |
| EMX2 | Q04743 | Homeobox protein EMX2 (Empty spiracles homolog 2) (Empty spiracles-like protein 2) | 252 |
| 1433E | P62258 | 14-3-3 protein epsilon (14-3-3E) | 255 |
| NTF3 | P20783 | Neurotrophin-3 (NT-3) (HDNF) (Nerve growth factor 2) (NGF-2) (Neurotrophic factor) | 257 |
| NGN2 | Q9H2A3 | Neurogenin-2 (NGN-2) (Class A basic helix-loop-helix protein 8) (bHLHa8) (Protein atonal homolog 4) | 272 |
| CAPZB | P47756 | F-actin-capping protein subunit beta (CapZ beta) | 277 |
| GPM6A | P51674 | Neuronal membrane glycoprotein M6-a (M6a) | 278 |
| HES1 | Q14469 | Transcription factor HES-1 (Class B basic helix-loop-helix protein 39) (bHLHb39) (Hairy and enhancer of split 1) (Hairy homolog) (Hairy-like protein) (hHL) | 280 |
| LBX1 | P52954 | Transcription factor LBX1 (Ladybird homeobox protein homolog 1) | 281 |
| MARE3 | Q9UPY8 | Microtubule-associated protein RP/EB family member 3 (EB1 protein family member 3)(EBF3) (End-binding protein 3) (EB3) (RP3) | 281 |
| SIX1 | Q15475 | Homeobox protein SIX1 (Sine oculis homeobox homolog 1) | 284 |
| FBSP1 | P0C2W1 | F-box/SPRY domain-containing protein 1 (F-box only protein 45) (hFbxo45) | 286 |
| HMOX1 | P09601 | Heme oxygenase 1 (HO-1) | 288 |
| TLX3 | O43711 | T-cell leukemia homeobox protein 3 (Homeobox protein Hox-11L2) | 291 |
| CDK5 | Q00535 | Cyclin-dependent kinase 5 (EC 2.7.11.22) (Cell division protein kinase 5) (Serine/threonine-protein kinase PSSALRE) (Tau protein kinase II catalytic subunit) (TPKII catalytic subunit) | 292 |
| SDCB1 | O00560 | Syntenin-1 (Melanoma differentiation-associated protein 9) (MDA-9) (Pro-TGF-alpha cytoplasmic domain-interacting protein 18) (TACIP18) (Scaffold protein Pbp1) (Syndecan-binding protein 1) | 298 |
| PITX3 | O75364 | Pituitary homeobox 3 (Homeobox protein PITX3) (Paired-like homeodomain transcription factor 3) | 302 |
| CHIP | Q9UNE7 | E3 ubiquitin-protein ligase CHIP (EC 6.3.2.-) (Antigen NY-CO-7) (CLL-associated antigen KW-8) (Carboxy terminus of Hsp70-interacting protein) (STIP1 homology and U box-containing protein 1) | 303 |
| PEX2 | P28328 | Peroxisome biogenesis factor 2 (35 kDa peroxisomal membrane protein) (Peroxin-2) (Peroxisomal membrane protein 3) (Peroxisome assembly factor 1) (PAF-1) (RING finger protein 72) | 305 |
| CD5R1 | Q15078 | Cyclin-dependent kinase 5 activator 1 (CDK5 activator 1) (Cyclin-dependent kinase 5 regulatory subunit 1) (TPKII regulatory subunit) [Cleaved into: Cyclin-dependent kinase 5 activator 1, p35 (p35) Cyclin-dependent kinase 5 activator 1, p25 (p25) (Tau protein kinase II 23 kDa subunit) (p23)] | 307 |
| PHX2B | Q99453 | Paired mesoderm homeobox protein 2B (Neuroblastoma Phox)(PHOX2B homeodomain protein)(Paired-like homeobox2B) | 314 |
| APOE | P02649 | Apolipoprotein E (Apo-E) | 317 |
| GBLP | P63244 | Guanine nucleotide-binding protein subunit beta-2-like 1 (Cell proliferation-inducing gene 21 protein) (Guanine nucleotide-binding protein subunit beta-like protein 12.3) (Human lung cancer oncogene 7 protein) (HLC-7) (Receptor for activated C kinase) (Receptor of activated protein kinase C 1) (RACK1) [Cleaved into: Guanine nucleotide-binding protein subunit beta-2-like 1, N-terminally processed] | 317 |
| PITX2 | Q99697 | Pituitary homeobox 2 (ALL1-responsive protein ARP1) (Homeobox protein PITX2) (Paired-like homeodomain transcription factor 2) (RIEG bicoid-related homeobox transcription factor) (Solurshin) | 317 |
| APEX1 | P27695 | DNA-(apurinic or apyrimidinic site) lyase (APEX nuclease) (APEN) (Apurinic-apyrimidinic endonuclease 1) (AP endonuclease 1) (APE-1) (REF-1) (Redox factor-1) [Cleaved into: DNA-(apurinic or apyrimidinic site) lyase, mitochondrial] | 318 |
| NECD | Q99608 | Necdin | 321 |
| PEX7 | O00628 | Peroxisomal targeting signal 2 receptor (PTS2 receptor) (Peroxin-7) | 323 |
| FOXB1 | Q99853 | Forkhead box protein B1 (Transcription factor FKH-5) | 325 |
| AA1R | P30542 | Adenosine receptor A1 | 326 |
| BARH1 | Q9BZE3 | BarH-like 1 homeobox protein | 327 |
| NDF4 | Q9HD90 | Neurogenic differentiation factor 4 (NeuroD4) (Class A basic helix-loop-helix protein 4) (bHLHa4) (Protein atonal homolog 3) (ATH-3) (Atoh3) | 331 |
| NUDC | Q9Y266 | Nuclear migration protein nudC (Nuclear distribution protein C homolog) | 331 |
| EFNB2 | P52799 | Ephrin-B2 (EPH-related receptor tyrosine kinase ligand 5) (LERK-5) (HTK ligand) (HTK-L) | 333 |
| VAX1 | Q5SQQ9 | Ventral anterior homeobox 1 | 334 |
| EFNB3 | Q15768 | Ephrin-B3 (EPH-related receptor transmembrane ligand ELK-L3) (EPH-related receptor tyrosine kinase ligand 8) (LERK-8) | 340 |
| NDEL1 | Q9GZM8 | Nuclear distribution protein nudE-like 1 (Protein Nudel) (Mitosin-associated protein 1) | 345 |
| EFNB1 | P98172 | Ephrin-B1 (EFL-3) (ELK ligand) (ELK-L) (EPH-related receptor tyrosine kinase ligand 2) (LERK-2) | 346 |
| NDE1 | Q9NXR1 | Nuclear distribution protein nudE homolog 1 (NudE) | 346 |
| GBX2 | P52951 | Homeobox protein GBX-2 (Gastrulation and brain-specific homeobox protein 2) | 348 |
| ISL1 | P61371 | Insulin gene enhancer protein ISL-1 (Islet-1) | 349 |
| SG196 | Q9H5K3 | Protein O-mannose kinase (POMK)(Protein kinase-like protein SgK196) (Sugen kinase 196) | 350 |
| WNT4 | P56705 | Protein Wnt-4 | 351 |
| CXCR4 | P61073 | C-X-C chemokine receptor type 4 (CXC-R4) (CXCR-4) (FB22) (Fusin) (HM89) (LCR1) (Leukocyte-derived seven transmembrane domain receptor) (LESTR) (NPYRL) (Stromal cell-derived factor 1 receptor) (SDF-1 receptor) (CD antigen CD184) | 352 |
| BKRB1 | P46663 | B1 bradykinin receptor (B1R) (BK-1 receptor) | 353 |
| ATOH1 | Q92858 | Protein atonal homolog 1 (Class A basic helix-loop-helix protein 14) (bHLHa14) (Helix-loop-helix protein hATH-1) (hATH1) | 354 |
| WNT11 | O96014 | Protein Wnt-11 | 354 |
| CX3C1 | P49238 | CX3C chemokine receptor 1 (C-X3-C CKR-1) (CX3CR1) (Beta chemokine receptor-like 1) (CMK-BRL-1) (CMK-BRL1) (Fractalkine receptor) (G-protein coupled receptor 13) (V28) | 355 |
| WNT5B | Q9H1J7 | Protein Wnt-5b | 359 |
| CCR4 | P51679 | C-C chemokine receptor type 4 (C-C CKR-4) (CC-CKR-4) (CCR-4) (CCR4) (K5-5) (CD antigen CD194) | 360 |
| CNR2 | P34972 | Cannabinoid receptor 2 (CB-2) (CB2) (hCB2) (CX5) | 360 |
| MK01 | P28482 | Mitogen-activated protein kinase 1 (MAP kinase 1) (MAPK 1) (EC 2.7.11.24) (ERT1) (Extracellular signal-regulated kinase 2) (ERK-2) (MAP kinase isoform p42) (p42-MAPK) (Mitogen-activated protein kinase 2) (MAP kinase 2) (MAPK 2) | 360 |
| ADA | P00813 | Adenosine deaminase (EC 3.5.4.4) (Adenosine aminohydrolase) | 363 |
| LHX6 | Q9UPM6 | LIM/homeobox protein Lhx6 (LIM homeobox protein 6) (LIM/homeobox protein Lhx6.1) | 363 |
| CD5R2 | Q13319 | Cyclin-dependent kinase 5 activator 2 (CDK5 activator 2) (Cyclin-dependent kinase 5 regulatory subunit 2) (p39) (p39I) | 367 |
| SSR2 | P30874 | Somatostatin receptor type 2 (SS-2-R) (SS2-R) (SS2R) (SRIF-1) | 369 |
| KCC1A | Q14012 | Calcium/calmodulin-dependent protein kinase type 1 (CaM kinase I) (CaM-KI) (CaM kinase I alpha) (CaMKI-alpha) | 370 |
| NKX21 | P43699 | Homeobox protein Nkx-2.1 (Homeobox protein NK-2 homolog A) (Thyroid nuclear factor 1) (Thyroid transcription factor 1) (TTF-1) | 371 |
| GPER1 | Q99527 | G-protein coupled estrogen receptor 1 (Chemoattractant receptor-like 2) (Flow-induced endothelial G-protein coupled receptor 1) (FEG-1) (G protein-coupled estrogen receptor 1) (G-protein coupled receptor 30) (GPCR-Br) (IL8-related receptor DRY12) (Lymphocyte-derived G-protein coupled receptor) (LYGPR) (Membrane estrogen receptor) (mER) | 375 |
| WNT5A | P41221 | Protein Wnt-5a | 380 |
| CXA1 | P17302 | Gap junction alpha-1 protein (Connexin-43) (Cx43) (Gap junction 43 kDa heart protein) | 382 |
| S1PR1 | P21453 | Sphingosine 1-phosphate receptor 1 (S1P receptor 1) (S1P1) (Endothelial differentiation G-protein coupled receptor 1) (Sphingosine 1-phosphate receptor Edg-1) (S1P receptor Edg-1) (CD antigen CD363) | 382 |
| SAV1 | Q9H4B6 | Protein salvador homolog 1 (45 kDa WW domain protein) (hWW45) | 383 |
| NR2E1 | Q9Y466 | Nuclear receptor subfamily 2 group E member 1 (Nuclear receptor TLX) (Protein tailless homolog) (Tll) (hTll) | 385 |
| BARH2 | Q9NY43 | BarH-like 2 homeobox protein | 387 |
| ALKB1 | Q13686 | Alkylated DNA repair protein alkB homolog 1 (Alpha-ketoglutarate-dependent dioxygenase ABH1) (DNA lyase ABH1) (EC 4.2.99.18) (DNA oxidative demethylase ALKBH1) | 389 |
| TGFB1 | P01137 | Transforming growth factor beta-1 (TGF-beta-1) [Cleaved into: Latency-associated peptide (LAP)] | 390 |
| SOX1 | O00570 | Transcription factor SOX-1 | 391 |
| CREB3 | O43889 | Cyclic AMP-responsive element-binding protein 3 (CREB-3) (cAMP-responsive element-binding protein 3) (Leucine zipper protein) (Luman) (Transcription factor LZIP-alpha) [Cleaved into: Processed cyclic AMP-responsive element-binding protein 3 (N-terminal Luman) (Transcriptionally active form)] | 395 |
| ARC | Q7LC44 | Activity-regulated cytoskeleton-associated protein (ARC/ARG3.1) (Activity-regulated gene 3.1 protein homolog) (Arg3.1) | 396 |
| BMP2 | P12643 | Bone morphogenetic protein 2 (BMP-2) (Bone morphogenetic protein 2A) (BMP-2A) | 396 |
| LEF1 | Q9UJU2 | Lymphoid enhancer-binding factor 1 (LEF-1) (T cell-specific transcription factor 1-alpha) (TCF1-alpha) | 399 |
| GFRA3 | O60609 | GDNF family receptor alpha-3 (GDNF receptor alpha-3) (GDNFR-alpha-3) (GFR-alpha-3) | 400 |
| MNX1 | P50219 | Motor neuron and pancreas homeobox protein 1 (Homeobox protein HB9) | 401 |
| RILP | Q96NA2 | Rab-interacting lysosomal protein | 401 |
| LHX5 | Q9H2C1 | LIM/homeobox protein Lhx5 (LIM homeobox protein 5) | 402 |
| LMX1B | O60663 | LIM homeobox transcription factor 1-beta (LIM/homeobox protein 1.2) (LMX-1.2) (LIM/homeobox protein LMX1B) | 402 |
| PEX13 | Q92968 | Peroxisomal membrane protein PEX13 (Peroxin-13) | 403 |
| PTEN | P60484 | Phosphatidylinositol 3,4,5-trisphosphate 3-phosphatase and dual-specificity protein phosphatase PTEN (EC 3.1.3.16) (EC 3.1.3.48) (EC 3.1.3.67) (Mutated in multiple advanced cancers 1) (Phosphatase and tensin homolog) | 403 |
| LHX1 | P48742 | LIM/homeobox protein Lhx1 (LIM homeobox protein 1) (Homeobox protein Lim-1) (hLim-1) | 406 |
| BMP4 | P12644 | Bone morphogenetic protein 4 (BMP-4) (Bone morphogenetic protein 2B) (BMP-2B) | 408 |
| LIS1 | P43034 | Platelet-activating factor acetylhydrolase IB subunit alpha (Lissencephaly-1 protein) (LIS-1) (PAF acetylhydrolase 45 kDa subunit) (PAF-AH 45 kDa subunit) (PAF-AH alpha) (PAFAH alpha) | 410 |
| H6ST1 | O60243 | Heparan-sulfate 6-O-sulfotransferase 1 (HS6ST-1) | 411 |
| COT2 | P24468 | COUP transcription factor 2 (COUP-TF2) (Apolipoprotein A-I regulatory protein 1) (ARP-1) (COUP transcription factor II) (COUP-TF II) (Nuclear receptor subfamily 2 group F member 2) | 414 |
| TGFB2 | P61812 | Transforming growth factor beta-2 (TGF-beta-2) (BSC-1 cell growth inhibitor) (Cetermin) (Glioblastoma-derived T-cell suppressor factor) (G-TSF) (Polyergin) [Cleaved into: Latency-associated peptide (LAP)] | 414 |
| PO4F1 | Q01851 | POU domain, class 4, transcription factor 1 (Brain-specific homeobox/POU domain protein 3A) (Brain-3A) (Brn-3A) (Homeobox/POU domain protein RDC-1) (Oct-T1) | 419 |
| DYXC1 | Q8WXU2 | Dyslexia susceptibility 1 candidate gene 1 protein | 420 |
| GSK3B | P49841 | Glycogen synthase kinase-3 beta (GSK-3 beta) (Serine/threonine-protein kinase GSK3B) | 420 |
| PAX6 | P26367 | Paired box protein Pax-6 (Aniridia type II protein) (Oculorhombin) | 422 |
| COT1 | P10589 | COUP transcription factor 1 (COUP-TF1) (COUP transcription factor I) (COUP-TF I) (Nuclear receptor subfamily 2 group F member 1) (V-erbA-related protein 3) (EAR-3) | 423 |
| MK09 | P45984 | Mitogen-activated protein kinase 9 (MAP kinase 9) (MAPK 9) (EC 2.7.11.24) (JNK-55) (Stress-activated protein kinase 1a) (SAPK1a) (Stress-activated protein kinase JNK2) (c-Jun N-terminal kinase 2) | 424 |
| PAR1 | P25116 | Proteinase-activated receptor 1 (PAR-1) (Coagulation factor II receptor) (Thrombin receptor) | 425 |
| SMAD3 | P84022 | Mothers against decapentaplegic homolog 3 (MAD homolog 3) (Mad3) (Mothers against DPP homolog 3) (hMAD-3) (JV15-2) (SMAD family member 3) (SMAD 3) (Smad3) (hSMAD3) | 425 |
| CCKAR | P32238 | Cholecystokinin receptor type A (CCK-A receptor) (CCK-AR) (Cholecystokinin-1 receptor) (CCK1-R) | 428 |
| SGK1 | O00141 | Serine/threonine-protein kinase Sgk1 (EC 2.7.11.1) (Serum/glucocorticoid-regulated kinase 1) | 431 |
| AP2A | P05549 | Transcription factor AP-2-alpha (AP2-alpha) (AP-2 transcription factor) (Activating enhancer-binding protein 2-alpha) (Activator protein 2) (AP-2) | 437 |
| TICN1 | Q08629 | Testican-1 (Protein SPOCK) | 439 |
| DCX | O43602 | Neuronal migration protein doublecortin (Doublin) (Lissencephalin-X) (Lis-X) | 441 |
| CADM1 | Q9BY67 | Cell adhesion molecule 1 (Immunoglobulin superfamily member 4) (IgSF4) (Nectin-like protein 2) (NECL-2) (Spermatogenic immunoglobulin superfamily) (SgIgSF) (Synaptic cell adhesion molecule) (SynCAM) (Tumor suppressor in lung cancer 1) (TSLC-1) | 442 |
| EDNRB | P24530 | Endothelin B receptor (ET-B) (ET-BR) (Endothelin receptor non-selective type) | 442 |
| DRD2 | P14416 | D(2) dopamine receptor (Dopamine D2 receptor) | 443 |
| GATA3 | P23771 | Trans-acting T-cell-specific transcription factor GATA-3 (GATA-binding factor 3) | 443 |
| PO3F2 | P20265 | POU domain, class 3, transcription factor 2 (Brain-specific homeobox/POU domain protein 2) (Brain-2) (Brn-2) (Nervous system-specific octamer-binding transcription factor N-Oct-3)(Octamer-binding protein 7)(Oct-7)(Octamer-binding transcription factor 7) (OTF-7) | 443 |
| TBB2B | Q9BVA1 | Tubulin beta-2B chain | 445 |
| DRD1 | P21728 | D(1A) dopamine receptor (Dopamine D1 receptor) | 446 |
| TBX20 | Q9UMR3 | T-box transcription factor TBX20 (T-box protein 20) | 447 |
| MP2K5 | Q13163 | Dual specificity mitogen-activated protein kinase kinase 5 (MAP kinase kinase 5) (MAPKK 5) (MAPK/ERK kinase 5) (MEK 5) | 448 |
| TBB3 | Q13509 | Tubulin beta-3 chain (Tubulin beta-4 chain) (Tubulin beta-III) | 450 |
| PTK6 | Q13882 | Protein-tyrosine kinase 6 (EC 2.7.10.2) (Breast tumor kinase) (Tyrosine-protein kinase BRK) | 451 |
| ILK | Q13418 | Integrin-linked protein kinase (EC 2.7.11.1) (59 kDa serine/threonine-protein kinase) (ILK-1) (ILK-2) (p59ILK) | 452 |
| FEZF2 | Q8TBJ5 | Fez family zinc finger protein 2 (Forebrain embryonic zinc finger-like protein 2) (Zinc finger protein 312) (Zinc finger protein Fez-like) | 459 |
| SRG2C | P0DJJ0 | SLIT-ROBO Rho GTPase-activating protein 2C (SLIT-ROBO Rho GTPase activating protein 2 pseudogene 1) | 459 |
| TNR1B | P20333 | Tumor necrosis factor receptor superfamily member 1B (Tumor necrosis factor receptor 2) (TNF-R2) (Tumor necrosis factor receptor type II) (TNF-RII) (TNFR-II) (p75) (p80 TNF-alpha receptor) (CD antigen CD120b) (Etanercept) [Cleaved into: Tumor necrosis factor receptor superfamily member 1b, membrane form Tumor necrosis factor-binding protein 2 (TBP-2) (TBPII)] | 461 |
| SHH | Q15465 | Sonic hedgehog protein (SHH) (HHG-1) [Cleaved into: Sonic hedgehog protein N-product Sonic hedgehog protein C-product] | 462 |
| MK10 | P53779 | Mitogen-activated protein kinase 10 (MAP kinase 10) (MAPK 10) (EC 2.7.11.24) (MAP kinase p49 3F12) (Stress-activated protein kinase 1b) (SAPK1b) (Stress-activated protein kinase JNK3) (c-Jun N-terminal kinase 3) | 464 |
| GFRA1 | P56159 | GDNF family receptor alpha-1 (GDNF receptor alpha-1) (GDNFR-alpha-1) (GFR-alpha-1) (RET ligand 1) (TGF-beta-related neurotrophic factor receptor 1) | 465 |
| PSN1 | P49768 | Presenilin-1 (PS-1) (EC 3.4.23.-) (Protein S182) [Cleaved into: Presenilin-1 NTF subunit Presenilin-1 CTF subunit Presenilin-1 CTF12 (PS1-CTF12)] | 467 |
| SMAD2 | Q15796 | Mothers against decapentaplegic homolog 2 (MAD homolog 2) (Mothers against DPP homolog 2) (JV18-1) (Mad-related protein 2) (hMAD-2) (SMAD family member 2) (SMAD 2) (Smad2) (hSMAD2) | 467 |
| MMP1 | P03956 | Interstitial collagenase (EC 3.4.24.7) (Fibroblast collagenase) (Matrix metalloproteinase-1) (MMP-1) [Cleaved into: 22 kDa interstitial collagenase 27 kDa interstitial collagenase] | 469 |
| FEZF1 | A0PJY2 | Fez family zinc finger protein 1 (Zinc finger protein 312B) | 475 |
| DCDC2 | Q9UHG0 | Doublecortin domain-containing protein 2 (Protein RU2S) | 476 |
| PAX3 | P23760 | Paired box protein Pax-3 (HuP2) | 479 |
| AKT1 | P31749 | RAC-alpha serine/threonine-protein kinase (EC 2.7.11.1) (Protein kinase B) (PKB) (Protein kinase B alpha) (PKB alpha) (Proto-oncogene c-Akt) (RAC-PK-alpha) | 480 |
| 5HT2B | P41595 | 5-hydroxytryptamine receptor 2B (5-HT-2B) (5-HT2B) (Serotonin receptor 2B) | 481 |
| AKT2 | P31751 | RAC-beta serine/threonine-protein kinase (EC 2.7.11.1) (Protein kinase Akt-2) (Protein kinase B beta) (PKB beta) (RAC-PK-beta) | 481 |
| IRX5 | P78411 | Iroquois-class homeodomain protein IRX-5 (Homeodomain protein IRX-2A) (Homeodomain protein IRXB2) (Iroquois homeobox protein 5) | 483 |
| ANGT | P01019 | Angiotensinogen (Serpin A8) [Cleaved into: Angiotensin-1 (Angiotensin 1-10) (Angiotensin I) (Ang I) Angiotensin-2 (Angiotensin 1-8) (Angiotensin II) (Ang II) Angiotensin-3 (Angiotensin 2-8) (Angiotensin III) (Ang III) (Des-Asp[1]-angiotensin II) Angiotensin-4 (Angiotensin 3-8) (Angiotensin IV) (Ang IV) Angiotensin 1-9 Angiotensin 1-7 Angiotensin 1-5 Angiotensin 1-4] | 485 |
| KTNA1 | O75449 | Katanin p60 ATPase-containing subunit A1 (Katanin p60 subunit A1) (EC 3.6.4.3) (p60 katanin) | 491 |
| FBLN3 | Q12805 | EGF-containing fibulin-like extracellular matrix protein 1 (Extracellular protein S1-5) (Fibrillin-like protein) (Fibulin-3) (FIBL-3) | 493 |
| PO3F3 | P20264 | POU domain, class 3, transcription factor 3 (Brain-specific homeobox/POU domain protein 1) (Brain-1) (Brn-1) (Octamer-binding protein 8) (Oct-8) (Octamer-binding transcription factor 8) (OTF-8) | 500 |
| ACVL1 | P37023 | Serine/threonine-protein kinase receptor R3 (SKR3) (EC 2.7.11.30) (Activin receptor-like kinase 1) (ALK-1) (TGF-B superfamily receptor type I) (TSR-I) | 503 |
| TGFR1 | P36897 | TGF-beta receptor type-1 (TGFR-1) (EC 2.7.11.30) (Activin A receptor type II-like protein kinase of 53kD) (Activin receptor-like kinase 5) (ALK-5) (ALK5) (Serine/threonine-protein kinase receptor R4) (SKR4) (TGF-beta type I receptor) (Transforming growth factor-beta receptor type I) (TGF-beta receptor type I) (TbetaR-I) | 503 |
| ONEC2 | O95948 | One cut domain family member 2 (Hepatocyte nuclear factor 6-beta) (HNF-6-beta) (One cut homeobox 2) (Transcription factor ONECUT-2) (OC-2) | 504 |
| YAP1 | P46937 | Yorkie homolog (65 kDa Yes-associated protein) (YAP65) | 504 |
| ACV1B | P36896 | Activin receptor type-1B (EC 2.7.11.30) (Activin receptor type IB) (ACTR-IB) (Activin receptor-like kinase 4) (ALK-4) (Serine/threonine-protein kinase receptor R2) (SKR2) | 505 |
| LAT1 | Q01650 | Large neutral amino acids transporter small subunit 1 (4F2 light chain) (4F2 LC) (4F2LC) (CD98 light chain) (Integral membrane protein E16) (L-type amino acid transporter 1) (hLAT1) (Solute carrier family 7 member 5) (y+ system cationic amino acid transporter) | 507 |
| SRF | P11831 | Serum response factor (SRF) | 508 |
| SHB | Q15464 | SH2 domain-containing adapter protein B | 509 |
| LYN | P07948 | Tyrosine-protein kinase Lyn (EC 2.7.10.2) (Lck/Yes-related novel protein tyrosine kinase) (V-yes-1 Yamaguchi sarcoma viral related oncogene homolog) (p53Lyn) (p56Lyn) | 512 |
| PNKP | Q96T60 | Bifunctional polynucleotide phosphatase/kinase (DNA 5'-kinase/3'-phosphatase) (Polynucleotide kinase-3'-phosphatase) [Includes: Polynucleotide 3'-phosphatase (2'(3')-polynucleotidase) Polynucleotide 5'-hydroxyl-kinase | 521 |
| AMGO2 | Q86SJ2 | Amphoterin-induced protein 2 (AMIGO-2) (Alivin-1) (Differentially expressed in gastric adenocarcinomas) (DEGA) | 522 |
| KS6B1 | P23443 | Ribosomal protein S6 kinase beta-1 (S6K-beta-1) (S6K1)(70 kDa ribosomal protein S6 kinase 1) (P70S6K1) (p70-S6K 1) (Ribosomal protein S6 kinase I) (Serine/threonine-protein kinase 14A) (p70 ribosomal S6 kinase alpha) (p70 S6 kinase alpha) (p70 S6KA) | 525 |
| ESR2 | Q92731 | Estrogen receptor beta (ER-beta) (Nuclear receptor subfamily 3 group A member 2) | 530 |
| NSMF | Q6X4W1 | NMDA receptor synaptonuclear signaling and neuronal migration factor (Nasal embryonic luteinizing hormone-releasing hormone factor) (Nasal embryonic LHRH factor) | 530 |
| TBX21 | Q9UL17 | T-box transcription factor TBX21 (T-box protein 21) (T-cell-specific T-box transcription factor T-bet) (Transcription factor TBLYM) | 535 |
| SRC | P12931 | Proto-oncogene tyrosine-protein kinase Src (EC 2.7.10.2) (Proto-oncogene c-Src) (pp60c-src) (p60-Src) | 536 |
| FYN | P06241 | Tyrosine-protein kinase Fyn (Proto-oncogene Syn) (Proto-oncogene c-Fyn) (Src-like kinase) (SLK) (p59-Fyn) | 537 |
| SRC8 | Q14247 | Src substrate cortactin (Amplaxin) (Oncogene EMS1) | 550 |
| HEXB | P07686 | Beta-hexosaminidase subunit beta (Beta-N-acetylhexosaminidase subunit beta) (Hexosaminidase subunit B) (Cervical cancer proto-oncogene 7 protein) (HCC-7) (N-acetyl-beta-glucosaminidase subunit beta) [Cleaved into: Beta-hexosaminidase subunit beta chain B Beta-hexosaminidase subunit beta chain A] | 556 |
| PDPK1 | O15530 | 3-phosphoinositide-dependent protein kinase 1 (hPDK1) (EC 2.7.11.1) | 556 |
| PAK3 | O75914 | Serine/threonine-protein kinase PAK 3 (EC 2.7.11.1) (Beta-PAK) (Oligophrenin-3) (p21-activated kinase 3) (PAK-3) | 559 |
| ARX | Q96QS3 | Homeobox protein ARX (Aristaless-related homeobox) | 562 |
| TPA | P00750 | Tissue-type plasminogen activator (t-PA) (t-plasminogen activator) (tPA) (EC 3.4.21.68) (Alteplase) (Reteplase) [Cleaved into: Tissue-type plasminogen activator chain A Tissue-type plasminogen activator chain B] | 562 |
| NDNF | Q8TB73 | Protein NDNF (Neuron-derived neurotrophic factor) | 568 |
| DPYL3 | Q14195 | Dihydropyrimidinase-related protein 3 (DRP-3) (Collapsin response mediator protein 4) (CRMP-4) (Unc-33-like phosphoprotein 1) (ULIP-1) | 570 |
| DPYL1 | Q14194 | Dihydropyrimidinase-related protein 1 (DRP-1) (Collapsin response mediator protein 1) (CRMP-1) (Unc-33-like phosphoprotein 3) (ULIP-3) | 572 |
| DPYL2 | Q16555 | Dihydropyrimidinase-related protein 2 (DRP-2) (Collapsin response mediator protein 2) (CRMP-2) (N2A3) (Unc-33-like phosphoprotein 2) (ULIP-2) | 572 |
| DPYL4 | O14531 | Dihydropyrimidinase-related protein 4 (DRP-4) (Collapsin response mediator protein 3) (CRMP-3) (UNC33-like phosphoprotein 4) (ULIP-4) | 572 |
| IF2B1 | Q9NZI8 | Insulin-like growth factor 2 mRNA-binding protein 1 (IGF2 mRNA-binding protein 1) (IMP-1) (IMP1) (Coding region determinant-binding protein) (CRD-BP) (IGF-II mRNA-binding protein 1) (VICKZ family member 1) (Zipcode-binding protein 1) (ZBP-1) | 577 |
| SHC1 | P29353 | SHC-transforming protein 1 (SHC-transforming protein 3) (SHC-transforming protein A) (Src homology 2 domain-containing-transforming protein C1) (SH2 domain protein C1) | 583 |
| DAB1 | O75553 | Disabled homolog 1 | 588 |
| KPCZ | Q05513 | Protein kinase C zeta type (EC 2.7.11.13) (nPKC-zeta) | 592 |
| KPCI | P41743 | Protein kinase C iota type (Atypical protein kinase C-lambda/iota) (PRKC-lambda/iota) (aPKC-lambda/iota) (nPKC-iota) | 596 |
| NR4A2 | P43354 | Nuclear receptor subfamily 4 group A member 2 (Immediate-early response protein NOT) (Orphan nuclear receptor NURR1) (Transcriptionally-inducible nuclear receptor) | 598 |
| NET1 | O95631 | Netrin-1 (Epididymis tissue protein Li 131P) | 604 |
| PGH2 | P35354 | Prostaglandin G/H synthase 2 (EC 1.14.99.1) (Cyclooxygenase-2) (COX-2) (PHS II) (Prostaglandin H2 synthase 2) (PGH synthase 2) (PGHS-2) (Prostaglandin-endoperoxide synthase 2) | 604 |
| MAG | P20916 | Myelin-associated glycoprotein (Siglec-4a) | 626 |
| PEX5 | P50542 | Peroxisomal targeting signal 1 receptor (PTS1 receptor) (PTS1R) (PTS1-BP) (Peroxin-5) (Peroxisomal C-terminal targeting signal import receptor) (Peroxisome receptor 1) | 639 |
| NRG1 | Q02297 | Pro-neuregulin-1, membrane-bound isoform (Pro-NRG1) [Cleaved into: Neuregulin-1 (Acetylcholine receptor-inducing activity) (ARIA) (Breast cancer cell differentiation factor p45) (Glial growth factor) (Heregulin) (HRG) (Neu differentiation factor) (Sensory and motor neuron-derived factor)] | 640 |
| LIMK1 | P53667 | LIM domain kinase 1 (LIMK-1) (EC 2.7.11.1) | 647 |
| RAF1 | P04049 | RAF proto-oncogene serine/threonine-protein kinase (EC 2.7.11.1) (Proto-oncogene c-RAF) (cRaf) (Raf-1) | 648 |
| DREB | Q16643 | Drebrin (Developmentally-regulated brain protein) | 649 |
| TRI32 | Q13049 | E3 ubiquitin-protein ligase TRIM32 (72 kDa Tat-interacting protein) (Tripartite motif-containing protein 32) (Zinc finger protein HT2A) | 653 |
| KTNB1 | Q9BVA0 | Katanin p80 WD40 repeat-containing subunit B1 (Katanin p80 subunit B1) (p80 katanin) | 655 |
| PMGT1 | Q8WZA1 | Protein O-linked-mannose beta-1,2-N-acetylglucosaminyltransferase 1 (POMGnT1) (EC 2.4.1.-) (UDP-GlcNAc:alpha-D-mannoside beta-1,2-N-acetylglucosaminyltransferase I.2) (GnT I.2) | 660 |
| LMNA | P02545 | Prelamin-A/C [Cleaved into: Lamin-A/C (70 kDa lamin) (Renal carcinoma antigen NY-REN-32)] | 664 |
| SH3K1 | Q96B97 | SH3 domain-containing kinase-binding protein 1 (CD2-binding protein 3) (CD2BP3) (Cbl-interacting protein of 85 kDa) (Human Src family kinase-binding protein 1) (HSB-1) | 665 |
| FZD3 | Q9NPG1 | Frizzled-3 (Fz-3) (hFz3) | 666 |
| KGP1 | Q13976 | cGMP-dependent protein kinase 1 (cGK 1) (cGK1) (EC 2.7.11.12) (cGMP-dependent protein kinase I) (cGKI) | 671 |
| KPCA | P17252 | Protein kinase C alpha type (PKC-A) (PKC-alpha) (EC 2.7.11.13) | 672 |
| KALM | P23352 | Anosmin-1 (Adhesion molecule-like X-linked) (Kallmann syndrome protein) | 680 |
| GPR56 | Q9Y653 | G-protein coupled receptor 56 (Protein TM7XN1) [Cleaved into: GPR56 N-terminal fragment (GPR56 NT) (GPR56(N)) (GPR56 extracellular subunit) (GPR56 subunit alpha) GPR56 C-terminal fragment (GPR56 CT) (GPR56(C)) (GPR56 seven-transmembrane subunit) (GPR56 7TM) (GPR56 subunit beta)] | 693 |
| CAPR1 | Q14444 | Caprin-1 (Cell cycle-associated protein 1) (Cytoplasmic activation- and proliferation-associated protein 1) (GPI-anchored membrane protein 1)(GPI-anchored protein p137)(GPI-p137)(Membrane component chromosome 11 surface marker 1)(RNA granule protein 105) | 709 |
| APBB1 | O00213 | Amyloid beta A4 precursor protein-binding family B member 1 (Protein Fe65) | 710 |
| CAD13 | P55290 | Cadherin-13 (Heart cadherin) (H-cadherin) (P105) (Truncated cadherin) (T-cad) (T-cadherin) | 713 |
| SUN2 | Q9UH99 | SUN domain-containing protein 2 (Protein unc-84 homolog B) (Rab5-interacting protein) (Rab5IP) (Sad1/unc-84 protein-like 2) | 717 |
| HOOK3 | Q86VS8 | Protein Hook homolog 3 (h-hook3) (hHK3) | 718 |
| GAS6 | Q14393 | Growth arrest-specific protein 6 (GAS-6) (AXL receptor tyrosine kinase ligand) | 721 |
| DTL | Q9NZJ0 | Denticleless protein homolog (DDB1- and CUL4-associated factor 2) (Lethal(2) denticleless protein homolog) (Retinoic acid-regulated nuclear matrix-associated protein) | 730 |
| HS90A | P07900 | Heat shock protein HSP 90-alpha (Heat shock 86 kDa) (HSP 86) (HSP86) (Renal carcinoma antigen NY-REN-38) | 732 |
| SATB2 | Q9UPW6 | DNA-binding protein SATB2 (Special AT-rich sequence-binding protein 2) | 733 |
| DNER | Q8NFT8 | Delta and Notch-like epidermal growth factor-related receptor | 737 |
| KPCE | Q02156 | Protein kinase C epsilon type (EC 2.7.11.13) (nPKC-epsilon) | 737 |
| PROX1 | Q92786 | Prospero homeobox protein 1 (Homeobox prospero-like protein PROX1) (PROX-1) | 737 |
| DCLK1 | O15075 | Serine/threonine-protein kinase DCLK1 (EC 2.7.11.1) (Doublecortin domain-containing protein 3A) (Doublecortin-like and CAM kinase-like 1) (Doublecortin-like kinase 1) | 740 |
| POMT1 | Q9Y6A1 | Protein O-mannosyl-transferase 1 (EC 2.4.1.109) (Dolichyl-phosphate-mannose--protein mannosyltransferase 1) | 747 |
| APBB2 | Q92870 | Amyloid beta A4 precursor protein-binding family B member 2 (Protein Fe65-like 1) | 758 |
| TAU | P10636 | Microtubule-associated protein tau (Neurofibrillary tangle protein) (Paired helical filament-tau) (PHF-tau) | 758 |
| DYR1A | Q13627 | Dual specificity tyrosine-phosphorylation-regulated kinase 1A (EC 2.7.12.1) (Dual specificity YAK1-related kinase) (HP86) (Protein kinase minibrain homolog) (MNBH) (hMNB) | 763 |
| CUL3 | Q13618 | Cullin-3 (CUL-3) | 768 |
| SEM3A | Q14563 | Semaphorin-3A (Semaphorin III) (Sema III) | 771 |
| KIRR3 | Q8IZU9 | Kin of IRRE-like protein 3 (Kin of irregular chiasm-like protein 3) (Nephrin-like protein 2) | 778 |
| CTNB1 | P35222 | Catenin beta-1 (Beta-catenin) | 781 |
| SIX4 | Q9UIU6 | Homeobox protein SIX4 (Sine oculis homeobox homolog 4) | 781 |
| RIN1 | Q13671 | Ras and Rab interactor 1 (Ras inhibitor JC99) (Ras interaction/interference protein 1) | 783 |
| SEM3F | Q13275 | Semaphorin-3F (Sema III/F) (Semaphorin IV) (Sema IV) | 785 |
| SMO | Q99835 | Smoothened homolog (SMO) (Protein Gx) | 787 |
| MARK2 | Q7KZI7 | Serine/threonine-protein kinase MARK2 (EC 2.7.11.1) (EC 2.7.11.26) (ELKL motif kinase 1) (EMK-1) (MAP/microtubule affinity-regulating kinase 2) (PAR1 homolog) (PAR1 homolog b) (Par-1b) (Par1b) | 788 |
| MGT5B | Q3V5L5 | Alpha-1,6-mannosylglycoprotein 6-beta-N-acetylglucosaminyltransferase B (EC 2.4.1.-) (EC 2.4.1.155) (Alpha-mannoside beta-1,6-N-acetylglucosaminyltransferase B) (GlcNAc-T Vb) (GNT-Vb) (hGnTVb) (Mannoside acetylglucosaminyltransferase 5B) (N-acetylglucosaminyl-transferase Vb) (N-acetylglucosaminyltransferase IX) (GNT-IX) | 792 |
| MARK1 | Q9P0L2 | Serine/threonine-protein kinase MARK1 (EC 2.7.11.1) (EC 2.7.11.26) (MAP/microtubule affinity-regulating kinase 1) (PAR1 homolog c) (Par-1c) (Par1c) | 795 |
| ITB1 | P05556 | Integrin beta-1 (Fibronectin receptor subunit beta) (Glycoprotein IIa) (GPIIA) (VLA-4 subunit beta) (CD antigen CD29) | 798 |
| ARHG7 | Q14155 | Rho guanine nucleotide exchange factor 7 (Beta-Pix) (COOL-1) (PAK-interacting exchange factor beta) (p85) | 803 |
| SUN1 | O94901 | SUN domain-containing protein 1 (Protein unc-84 homolog A) (Sad1/unc-84 protein-like 1) | 812 |
| FGFR2 | P21802 | Fibroblast growth factor receptor 2 (FGFR-2) (K-sam) (KGFR) (Keratinocyte growth factor receptor) (CD antigen CD332) | 821 |
| FER | P16591 | Tyrosine-protein kinase Fer (Feline encephalitis virus-related kinase FER) (Fujinami poultry sarcoma/Feline sarcoma-related protein Fer) (Proto-oncogene c-Fer) (Tyrosine kinase 3) (p94-Fer) | 822 |
| FES | P07332 | Tyrosine-protein kinase Fes/Fps (Feline sarcoma/Fujinami avian sarcoma oncogene homolog) (Proto-oncogene c-Fes) (Proto-oncogene c-Fps) (p93c-fes) | 822 |
| FGFR1 | P11362 | Fibroblast growth factor receptor 1 (FGFR-1) (EC 2.7.10.1) (Basic fibroblast growth factor receptor 1) (BFGFR) (bFGF-R-1) (Fms-like tyrosine kinase 2) (FLT-2) (N-sam) (Proto-oncogene c-Fgr) (CD antigen CD331) | 822 |
| NTRK2 | Q16620 | BDNF/NT-3 growth factors receptor (EC 2.7.10.1) (GP145-TrkB) (Trk-B) (Neurotrophic tyrosine kinase receptor type 2) (TrkB tyrosine kinase) (Tropomyosin-related kinase B) | 822 |
| ADAM8 | P78325 | Disintegrin and metalloproteinase domain-containing protein 8 (ADAM 8)(Cell surface antigen MS2) (CD antigen CD156a) | 824 |
| HIF1A | Q16665 | Hypoxia-inducible factor 1-alpha (HIF-1-alpha) (HIF1-alpha) (ARNT-interacting protein) (Basic-helix-loop-helix-PAS protein MOP1) (Class E basic helix-loop-helix protein 78) (bHLHe78) (Member of PAS protein 1) (PAS domain-containing protein 8) | 826 |
| SORT | Q99523 | Sortilin (100 kDa NT receptor) (Glycoprotein 95) (Gp95) (Neurotensin receptor 3) (NT3) (NTR3) | 831 |
| NTRK3 | Q16288 | NT-3 growth factor receptor (EC 2.7.10.1) (GP145-TrkC) (Trk-C) (Neurotrophic tyrosine kinase receptor type 3) (TrkC tyrosine kinase) | 839 |
| DISC1 | Q9NRI5 | Disrupted in schizophrenia 1 protein | 854 |
| PDC6I | Q8WUM4 | Programmed cell death 6-interacting protein (PDCD6-interacting protein) (ALG-2-interacting protein 1) (ALG-2-interacting protein X) (Hp95) | 868 |
| CADH1 | P12830 | Cadherin-1 (CAM 120/80) (Epithelial cadherin) (E-cadherin) (Uvomorulin) (CD antigen CD324) [Cleaved into: E-Cad/CTF1 E-Cad/CTF2 E-Cad/CTF3] | 882 |
| TYRO3 | Q06418 | Tyrosine-protein kinase receptor TYRO3 (EC 2.7.10.1) (Tyrosine-protein kinase BYK) (Tyrosine-protein kinase DTK) (Tyrosine-protein kinase RSE) (Tyrosine-protein kinase SKY) (Tyrosine-protein kinase TIF) | 890 |
| UFO | P30530 | Tyrosine-protein kinase receptor UFO (EC 2.7.10.1) (AXL oncogene) | 894 |
| CADH2 | P19022 | Cadherin-2 (CDw325) (Neural cadherin) (N-cadherin) (CD antigen CD325) | 906 |
| KPCD1 | Q15139 | Serine/threonine-protein kinase D1 (EC 2.7.11.13) (Protein kinase C mu type) (Protein kinase D) (nPKC-D1) (nPKC-mu) | 912 |
| NRP1 | O14786 | Neuropilin-1 (Vascular endothelial cell growth factor 165 receptor) (CD antigen CD304) | 923 |
| NRP2 | O60462 | Neuropilin-2 (Vascular endothelial cell growth factor 165 receptor 2) | 931 |
| UNC5C | O95185 | Netrin receptor UNC5C (Protein unc-5 homolog 3) (Protein unc-5 homolog C) | 931 |
| MDGA1 | Q8NFP4 | MAM domain-containing glycosylphosphatidylinositol anchor protein 1 (GPI and MAM protein) (GPIM) (Glycosylphosphatidylinositol-MAM) (MAM domain-containing protein 3) | 955 |
| MATN2 | O00339 | Matrilin-2 | 956 |
| EPHA2 | P29317 | Ephrin type-A receptor 2 (Epithelial cell kinase) (Tyrosine-protein kinase receptor ECK) | 976 |
| EPHA3 | P29320 | Ephrin type-A receptor 3 (EPH-like kinase 4) (EK4) (hEK4) (HEK) (Human embryo kinase) (Tyrosine-protein kinase TYRO4) (Tyrosine-protein kinase receptor ETK1) (Eph-like tyrosine kinase 1) | 983 |
| EPHB1 | P54762 | Ephrin type-B receptor 1 (ELK) (EPH tyrosine kinase 2) (EPH-like kinase 6) (EK6) (hEK6) (Neuronally-expressed EPH-related tyrosine kinase) (NET) (Tyrosine-protein kinase receptor EPH-2) | 984 |
| EPHA4 | P54764 | Ephrin type-A receptor 4 (EPH-like kinase 8) (EK8) (hEK8)(Tyrosine-protein kinase TYRO1)(Tyrosine-protein kinase receptor SEK) | 986 |
| EPHB3 | P54753 | Ephrin type-B receptor 3 (EPH-like tyrosine kinase 2) (EPH-like kinase 2) (Embryonic kinase 2) (EK2) (hEK2) (Tyrosine-protein kinase TYRO6) | 998 |
| EPHA8 | P29322 | Ephrin type-A receptor 8 (EPH- and ELK-related kinase) (EPH-like kinase 3) (EK3) (hEK3) (Tyrosine-protein kinase receptor EEK) | 1005 |
| FAK2 | Q14289 | Protein-tyrosine kinase 2-beta (Calcium-dependent tyrosine kinase) (CADTK) (Calcium-regulated non-receptor proline-rich tyrosine kinase) (Cell adhesion kinase beta) (CAK-beta) (CAKB) (Focal adhesion kinase 2) (FADK 2) (Proline-rich tyrosine kinase 2) (Related adhesion focal tyrosine kinase) (RAFTK) | 1009 |
| ODO1 | Q02218 | 2-oxoglutarate dehydrogenase, mitochondrial (EC 1.2.4.2) (2-oxoglutarate dehydrogenase complex component E1) (OGDC-E1) (Alpha-ketoglutarate dehydrogenase) | 1023 |
| CDKL5 | O76039 | Cyclin-dependent kinase-like 5 (EC 2.7.11.22) (Serine/threonine-protein kinase 9) | 1030 |
| SEM6A | Q9H2E6 | Semaphorin-6A (Semaphorin VIA) (Sema VIA) (Semaphorin-6A-1) (SEMA6A-1) | 1030 |
| ACK1 | Q07912 | Activated CDC42 kinase 1 (ACK-1) (EC 2.7.10.2) (EC 2.7.11.1) (Tyrosine kinase non-receptor protein 2) | 1038 |
| CNTN2 | Q02246 | Contactin-2 (Axonal glycoprotein TAG-1) (Axonin-1) (Transient axonal glycoprotein 1) (TAX-1) | 1040 |
| ULK1 | O75385 | Serine/threonine-protein kinase ULK1 (Autophagy-related protein 1 homolog) (ATG1) (hATG1) (Unc-51-like kinase 1) | 1050 |
| ITA3 | P26006 | Integrin alpha-3 (CD49 antigen-like family member C) (FRP-2) (Galactoprotein B3) (GAPB3) (VLA-3 subunit alpha) (CD antigen CD49c) [Cleaved into: Integrin alpha-3 heavy chain Integrin alpha-3 light chain] | 1051 |
| FAK1 | Q05397 | Focal adhesion kinase 1 (FADK 1) (Focal adhesion kinase-related nonkinase) (FRNK) (Protein phosphatase 1 regulatory subunit 71) (PPP1R71) (Protein-tyrosine kinase 2) (p125FAK) (pp125FAK) | 1052 |
| SRCN1 | Q9C0H9 | SRC kinase signaling inhibitor 1 (SNAP-25-interacting protein) (SNIP) (p130Cas-associated protein) (p140Cap) | 1055 |
| PTK7 | Q13308 | Inactive tyrosine-protein kinase 7 (Colon carcinoma kinase 4) (CCK-4) (Protein-tyrosine kinase 7) (Pseudo tyrosine kinase receptor 7) (Tyrosine-protein kinase-like 7) | 1070 |
| SRGP2 | O75044 | SLIT-ROBO Rho GTPase-activating protein 2 (srGAP2) (Formin-binding protein 2) (Rho GTPase-activating protein 34) | 1071 |
| K0319 | Q5VV43 | Dyslexia-associated protein KIAA0319 | 1072 |
| SRGP1 | Q7Z6B7 | SLIT-ROBO Rho GTPase-activating protein 1 (srGAP1) (Rho GTPase-activating protein 13) | 1085 |
| SRGP3 | O43295 | SLIT-ROBO Rho GTPase-activating protein 3 (srGAP3) (Mental disorder-associated GAP) (Rho GTPase-activating protein 14) (WAVE-associated Rac GTPase-activating protein) (WRP) | 1099 |
| RET | P07949 | Proto-oncogene tyrosine-protein kinase receptor Ret (EC 2.7.10.1) (Cadherin family member 12) (Proto-oncogene c-Ret) [Cleaved into: Soluble RET kinase fragment Extracellular cell-membrane anchored RET cadherin 120 kDa fragment] | 1114 |
| JAK2 | O60674 | Tyrosine-protein kinase JAK2 (EC 2.7.10.2) (Janus kinase 2) (JAK-2) | 1132 |
| ANS1A | Q92625 | Ankyrin repeat and SAM domain-containing protein 1A (Odin) | 1134 |
| MYO1B | O43795 | Unconventional myosin-Ib (MYH-1c) (Myosin I alpha) (MMI-alpha) (MMIa) | 1136 |
| TSC1 | Q92574 | Hamartin (Tuberous sclerosis 1 protein) | 1164 |
| ABL2 | P42684 | Abelson tyrosine-protein kinase 2 (Abelson murine leukemia viral oncogene homolog 2) (Abelson-related gene protein) | 1182 |
| DAB2P | Q5VWQ8 | Disabled homolog 2-interacting protein (DAB2 interaction protein) (DAB2-interacting protein) (ASK-interacting protein 1) (AIP-1) (DOC-2/DAB-2 interactive protein) | 1189 |
| ATN1 | P54259 | Atrophin-1 (Dentatorubral-pallidoluysian atrophy protein) | 1190 |
| RTN4 | Q9NQC3 | Reticulon-4 (Foocen) (Neurite outgrowth inhibitor) (Nogo protein) (Neuroendocrine-specific protein) (NSP) (Neuroendocrine-specific protein C homolog) (RTN-x) (Reticulon-5) | 1192 |
| CHL1 | O00533 | Neural cell adhesion molecule L1-like protein (Close homolog of L1) [Cleaved into: Processed neural cell adhesion molecule L1-like protein] | 1208 |
| ZSWM6 | Q9HCJ5 | Zinc finger SWIM domain-containing protein 6 | 1215 |
| JAG1 | P78504 | Protein jagged-1 (Jagged1) (hJ1) (CD antigen CD339) | 1218 |
| ERBB2 | P04626 | Receptor tyrosine-protein kinase erbB-2 (Metastatic lymph node gene 19 protein) (MLN 19) (Proto-oncogene Neu) (Proto-oncogene c-ErbB-2) (Tyrosine kinase-type cell surface receptor HER2) (p185erbB2) (CD antigen CD340) | 1255 |
| L1CAM | P32004 | Neural cell adhesion molecule L1 (N-CAM-L1) (NCAM-L1) (CD antigen CD171) | 1257 |
| DIAP1 | O60610 | Protein diaphanous homolog 1 (Diaphanous-related formin-1) (DRF1) | 1272 |
| DCTN1 | Q14203 | Dynactin subunit 1 (150 kDa dynein-associated polypeptide) (DAP-150) (DP-150) (p135) (p150-glued) | 1278 |
| MYO6 | Q9UM54 | Unconventional myosin-VI (Unconventional myosin-6) | 1294 |
| ASTN1 | O14525 | Astrotactin-1 | 1302 |
| NRCAM | Q92823 | Neuronal cell adhesion molecule (Nr-CAM) (Neuronal surface protein Bravo) (hBravo) (NgCAM-related cell adhesion molecule) (Ng-CAM-related) | 1304 |
| ERBB4 | Q15303 | Receptor tyrosine-protein kinase erbB-4 (Proto-oncogene-like protein c-ErbB-4) (Tyrosine kinase-type cell surface receptor HER4) (p180erbB4) [Cleaved into: ERBB4 intracellular domain (4ICD) (E4ICD) (s80HER4)] | 1308 |
| JIP4 | O60271 | C-Jun-amino-terminal kinase-interacting protein 4 (JIP-4) (JNK-interacting protein 4) (Cancer/testis antigen 89) (CT89) (Human lung cancer oncogene 6 protein) (HLC-6) (JNK-associated leucine-zipper protein) (JLP) (Mitogen-activated protein kinase 8-interacting protein 4) (Proliferation-inducing protein 6) (Protein highly expressed in testis) (PHET) (Sperm surface protein) (Sperm-associated antigen 9) (Sperm-specific protein) (Sunday driver 1) | 1321 |
| MINK1 | Q8N4C8 | Misshapen-like kinase 1 (EC 2.7.11.1) (GCK family kinase MiNK) (MAPK/ERK kinase kinase kinase 6) (MEK kinase kinase 6) (MEKKK 6) (Misshapen/NIK-related kinase) (Mitogen-activated protein kinase kinase kinase kinase 6) | 1332 |
| KANK1 | Q14678 | KN motif and ankyrin repeat domain-containing protein 1 (Ankyrin repeat domain-containing protein 15) (Kidney ankyrin repeat-containing protein) | 1352 |
| ROCK1 | Q13464 | Rho-associated protein kinase 1 (Renal carcinoma antigen NY-REN-35) (Rho-associated, coiled-coil-containing protein kinase 1) (Rho-associated, coiled-coil-containing protein kinase I) (ROCK-I) (p160 ROCK-1) (p160ROCK) | 1354 |
| TENR | Q92752 | Tenascin-R (TN-R) (Janusin) (Restrictin) | 1358 |
| IGF1R | P08069 | Insulin-like growth factor 1 receptor (Insulin-like growth factor I receptor) (IGF-I receptor) (CD antigen CD221) [Cleaved into: Insulin-like growth factor 1 receptor alpha chain Insulin-like growth factor 1 receptor beta chain] | 1367 |
| ROBO2 | Q9HCK4 | Roundabout homolog 2 | 1378 |
| ROBO3 | Q96MS0 | Roundabout homolog 3 (Roundabout-like protein 3) | 1386 |
| MET | P08581 | Hepatocyte growth factor receptor (HGF receptor) (HGF/SF receptor) (Proto-oncogene c-Met) (Scatter factor receptor) (SF receptor) (Tyrosine-protein kinase Met) | 1390 |
| PTPRK | Q15262 | Receptor-type tyrosine-protein phosphatase kappa (Protein-tyrosine phosphatase kappa) (R-PTP-kappa) (EC 3.1.3.48) | 1439 |
| DCC | P43146 | Netrin receptor DCC (Colorectal cancer suppressor) (Immunoglobulin superfamily DCC subclass member 1) (Tumor suppressor protein DCC) | 1447 |
| MAGI2 | Q86UL8 | Membrane-associated guanylate kinase, WW and PDZ domain-containing protein 2 (Atrophin-1-interacting protein 1) (AIP-1) (Atrophin-1-interacting protein A) (Membrane-associated guanylate kinase inverted 2) (MAGI-2) | 1455 |
| CO3A1 | P02461 | Collagen alpha-1(III) chain | 1466 |
| RPGF2 | Q9Y4G8 | Rap guanine nucleotide exchange factor 2 (Cyclic nucleotide ras GEF) (CNrasGEF) (Neural RAP guanine nucleotide exchange protein) (nRap GEP) (PDZ domain-containing guanine nucleotide exchange factor 1) (PDZ-GEF1) (RA-GEF-1) | 1499 |
| WDR62 | O43379 | WD repeat-containing protein 62 | 1518 |
| SLIT2 | O94813 | Slit homolog 2 protein (Slit-2) [Cleaved into: Slit homolog 2 protein N-product Slit homolog 2 protein C-product] | 1529 |
| SLIT1 | O75093 | Slit homolog 1 protein (Slit-1) (Multiple epidermal growth factor-like domains protein 4) (Multiple EGF-like domains protein 4) | 1534 |
| GLI3 | P10071 | Transcriptional activator GLI3 (GLI3 form of 190 kDa) (GLI3-190) (GLI3 full length protein) (GLI3FL) [Cleaved into: Transcriptional repressor GLI3R (GLI3 C-terminally truncated form) (GLI3 form of 83 kDa) (GLI3-83)] | 1580 |
| LRP6 | O75581 | Low-density lipoprotein receptor-related protein 6 (LRP-6) | 1613 |
| SHRM2 | Q13796 | Protein Shroom2 (Apical-like protein) (Protein APXL) | 1616 |
| ALK | Q9UM73 | ALK tyrosine kinase receptor (EC 2.7.10.1) (Anaplastic lymphoma kinase) (CD antigen CD246) | 1620 |
| TOP2B | Q02880 | DNA topoisomerase 2-beta (EC 5.99.1.3) (DNA topoisomerase II, beta isozyme) | 1626 |
| SCRIB | Q14160 | Protein scribble homolog (Scribble) (hScrib) (Protein LAP4) | 1630 |
| ROBO1 | Q9Y6N7 | Roundabout homolog 1 (Deleted in U twenty twenty) (H-Robo-1) | 1651 |
| LAMB1 | P07942 | Laminin subunit beta-1 (Laminin B1 chain) (Laminin-1 subunit beta) (Laminin-10 subunit beta) (Laminin-12 subunit beta) (Laminin-2 subunit beta) (Laminin-6 subunit beta) (Laminin-8 subunit beta) | 1786 |
| PLXB2 | O15031 | Plexin-B2 (MM1) | 1838 |
| GRDN | Q3V6T2 | Girdin (Akt phosphorylation enhancer) (APE) (Coiled-coil domain-containing protein 88A) (G alpha-interacting vesicle-associated protein) (GIV) (Girders of actin filament) (Hook-related protein 1) (HkRP1) | 1871 |
| PLXA3 | P51805 | Plexin-A3 (Plexin-4) (Semaphorin receptor SEX) | 1871 |
| NAV1 | Q8NEY1 | Neuron navigator 1 (Pore membrane and/or filament-interacting-like protein 3) (Steerin-1) (Unc-53 homolog 1) (unc53H1) | 1877 |
| MYH10 | P35580 | Myosin-10 (Cellular myosin heavy chain, type B) (Myosin heavy chain 10) (Myosin heavy chain, non-muscle IIb) (Non-muscle myosin heavy chain B) (NMMHC-B) (Non-muscle myosin heavy chain IIb) (NMMHC II-b) (NMMHC-IIB) | 1976 |
| PCM1 | Q15154 | Pericentriolar material 1 protein (PCM-1) (hPCM-1) | 2024 |
| SORL | Q92673 | Sortilin-related receptor (Low-density lipoprotein receptor relative with 11 ligand-binding repeats) (LDLR relative with 11 ligand-binding repeats) (LR11) (SorLA-1) (Sorting protein-related receptor containing LDLR class A repeats) (SorLA) | 2214 |
| RTTN | Q86VV8 | Rotatin | 2226 |
| CAC1B | Q00975 | Voltage-dependent N-type calcium channel subunit alpha-1B (Brain calcium channel III) (BIII) (Calcium channel, L type, alpha-1 polypeptide isoform 5) (Voltage-gated calcium channel subunit alpha Cav2.2) | 2339 |
| LRRK2 | Q5S007 | Leucine-rich repeat serine/threonine-protein kinase 2 (EC 2.7.11.1) (Dardarin) | 2527 |
| NOTC1 | P46531 | Neurogenic locus notch homolog protein 1 (Notch 1) (hN1) (Translocation-associated notch protein TAN-1) [Cleaved into: Notch 1 extracellular truncation Notch 1 intracellular domain (NICD)] | 2555 |
| FLNB | O75369 | Filamin-B (FLN-B) (ABP-278) (ABP-280 homolog) (Actin-binding-like protein) (Beta-filamin) (Filamin homolog 1) (Fh1) (Filamin-3) (Thyroid autoantigen) (Truncated actin-binding protein) (Truncated ABP) | 2602 |
| FLNA | P21333 | Filamin-A (FLN-A) (Actin-binding protein 280) (ABP-280) (Alpha-filamin) (Endothelial actin-binding protein) (Filamin-1) (Non-muscle filamin) | 2647 |
| NF1 | P21359 | Neurofibromin (Neurofibromatosis-related protein NF-1) [Cleaved into: Neurofibromin truncated] | 2839 |
| CELR2 | Q9HCU4 | Cadherin EGF LAG seven-pass G-type receptor 2 (Cadherin family member 10) (Epidermal growth factor-like protein 2) (EGF-like protein 2) (Flamingo homolog 3) (Multiple epidermal growth factor-like domains protein 3) (Multiple EGF-like domains protein 3) | 2923 |
| CELR1 | Q9NYQ6 | Cadherin EGF LAG seven-pass G-type receptor 1 (Cadherin family member 9) (Flamingo homolog 2) (hFmi2) | 3014 |
| CELR3 | Q9NYQ7 | Cadherin EGF LAG seven-pass G-type receptor 3 (Cadherin family member 11) (Epidermal growth factor-like protein 1) (EGF-like protein 1) (Flamingo homolog 1) (hFmi1) (Multiple EGF-like domains protein 2) | 3312 |
| PCNT | O95613 | Pericentrin (Kendrin) (Pericentrin-B) | 3336 |
| RELN | P78509 | Reelin | 3460 |
| ASPM | Q8IZT6 | Abnormal spindle-like microcephaly-associated protein (Abnormal spindle protein homolog) (Asp homolog) | 3477 |
| PGBM | P98160 | Basement membrane-specific heparan sulfate proteoglycan core protein (HSPG) (Perlecan) (PLC) [Cleaved into: Endorepellin LG3 peptide] | 4391 |
| LRP1 | Q07954 | Prolow-density lipoprotein receptor-related protein 1 (LRP-1) (Alpha-2-macroglobulin receptor) (A2MR) (Apolipoprotein E receptor) (APOER) (CD antigen CD91) [Cleaved into: Low-density lipoprotein receptor-related protein 1 85 kDa subunit (LRP-85) Low-density lipoprotein receptor-related protein 1 515 kDa subunit (LRP-515) Low-density lipoprotein receptor-related protein 1 intracellular domain (LRPICD)] | 4544 |
| DYHC1 | Q14204 | Cytoplasmic dynein 1 heavy chain 1 (Cytoplasmic dynein heavy chain 1) (Dynein heavy chain, cytosolic) | 4646 |
